# Manipulating the perception of time affects voluntary breath‐holding duration

**DOI:** 10.14814/phy2.14309

**Published:** 2019-12-12

**Authors:** Hannah J. Vigran, Anna G. Kapral, Eric D. Tytell, Mimi H. Kao

**Affiliations:** ^1^ Department of Biology Tufts University Medford Massachusetts

**Keywords:** breath holding, involuntary breathing movements, psychology, time perception

## Abstract

In this study, we examined how time perception, a psychological factor, impacts the physiological response to prolonged, voluntary breath holding. Participants (*n* = 26) held their breath while watching a distorted timer that made it appear as though time was moving up to 40% faster or slower than real time. We monitored total breath‐holding duration under different time manipulation conditions as well as the onset of involuntary breathing movements. This physiological breaking point marks the end of the “easy‐going” phase of apnea and the start of the “struggle” phase. Based on prior work showing that psychological factors, such as attention and motivation, can influence the length of the struggle phase, we hypothesized that manipulating the perception of time would affect overall breath‐holding duration by changing the duration of the struggle phase, but not the easy‐going phase. We found that time perception can be successfully manipulated using a distorted timekeeper, and total breath‐holding duration correlated with perceived time, not actual time. Contrary to our hypothesis, this effect was attributable to changes in the onset of the physiological breaking point, not changes in the length of the struggle phase. These results demonstrate that unconscious psychological factors and cognitive processes can significantly influence fundamental physiological processes.

## Graphical abstract

Time estimation is skewed following exposure to a distorted timer. Manipulation of time perception modulates the duration of voluntary breath holding by altering the onset of the physiological breaking point.

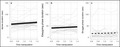

## 1 INTRODUCTION

Different people can voluntarily hold their breath for different amounts of time, depending on factors such as physical fitness, prior breath‐holding training, attention or distraction, and an individual's ability to withstand discomfort (Alpher, Nelson, & Blanton, 1986; Laurino et al., 2012; Lin, Lally, Moore, & Hong, 1974; Schagatay, Kampen, Emanuelsson, & Holm, 2000). Ultimately, breath‐hold duration is limited by physiological factors, including starting lung volume (Whitelaw, McBride, & Ford, 1987), metabolic rate and exercise (Ferretti, 2001), the decrease in blood oxygen levels (hypoxia) and the buildup of carbon dioxide (hypercapnia; Lin et al., 1974; Schagatay et al., 2000).

Although physiological factors clearly limit maximum breath‐holding duration, voluntary breath holding can be influenced by psychological factors, such as attention (Alpher et al., 1986), which suggests that cognitive processes may influence physiological processes. Recently, it has been shown that marine mammals trained to perform dives of different durations adjust their heart rate depending on the anticipated dive duration, demonstrating cognitive control of physiological responses to breath holding (Elmegaard, Johnson, Madsen, & McDonald, 2016). In this study, we manipulated a psychological factor, the perception of time, and measured its impact on the duration of voluntary breath holding and the onset of involuntary breathing movements, a physiological response.

**Figure 1:**
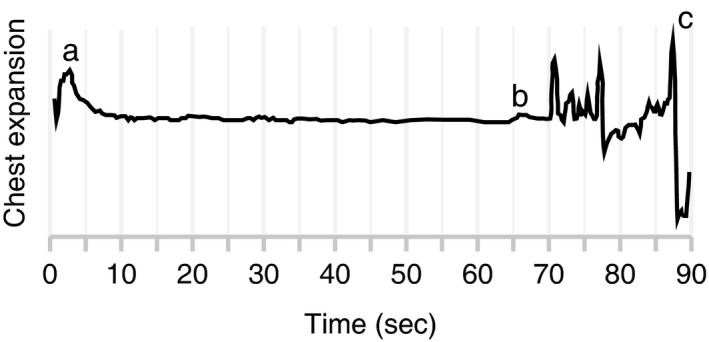
Example of thoracic movements during a breath hold (arbitrary units). Point a shows the chest expansion prior to beginning a breath hold. Point b indicates the onset of involuntary breathing movements, or the physiological breaking point. Point c indicates chest expansion at the termination of apnea. From Point a to Point b represents the duration of the “easy‐going” phase. From Point b to Point c represents the duration of the “struggle” phase

These involuntary breathing movements separate a breath hold into two phases: the “easy‐going” phase and the “struggle” phase (Figure 1) (Dejours, Puccinelli, Armand, & Dicharry, 1965; Schagatay et al., 2000). The easy‐going phase is the period during which it is easy for an individual to hold their breath. During this phase, there is little discomfort and respiratory muscle movement. The struggle phase begins at a point called the physiological breaking point, when small chest movements, the involuntary breathing movements, begin. These breathing movements, together with hypercapnia‐induced cerebral vasodilation and peripheral vasoconstriction, are thought to act together to increase blood flow to the heart and brain (Dujic et al., 2009).

Previous studies have suggested that the length of the easy‐going phase is determined mainly by physiological factors (Schagatay et al., 2000; Lin et al., 1974). In contrast, the duration of the struggle phase is thought to be determined primarily by psychological factors, including the willingness of subjects to resist the discomfort of involuntary breathing movements and the increasing urge to breathe (Alpher et al., 1986; Schagatay et al., 2000; Thompson‐Lake, De La Garza II, & Hajek, 2017).

In this study, we investigated the extent to which unconscious psychological factors can influence overall breath‐holding duration and the durations of the easy‐going and struggle phases. Specifically, we manipulated people's perception of time using a distorted timer that ran up to 40% faster or slower than real time. We hypothesized that this manipulation would affect the overall breath‐holding duration by changing the duration of the struggle phase, but not the easy‐going phase, because the struggle phase is known to be influenced by psychological factors. Instead, we found that the time manipulation affects the duration of the easy‐going phase, but not the struggle phase. Our results provide further support for the idea that cognitive processes can have substantial influence on fundamental physiological processes.

## 2 MATERIALS AND METHODS

### 2.1 Participants

All procedures were approved by the Tufts University Institutional Research Board (Protocol #1807027). Subjects were male (*n* = 18), female (*n* = 15), and unspecified (*n* = 1) Tufts University undergraduates (18–22 years old; mean 19.6 ± 1.3 (*SD*) years) who received compensation for their participation. Each participant completed a survey of demographic information, including age and gender.

A respiratory belt transducer (AD Instruments) was secured tightly around each participant's chest to detect chest movements. Data were recorded using PowerLab 6 and LabChart version 8 (ADInstruments; sampling rate = 1,000 Hz).

### 2.2 Time manipulation

We manipulated perception of time using a visual timer that appeared to indicate 10 s of elapsed time, but was actually sped up or slowed down. The time conditions included 0.6×, 0.8×, 1×, 1.2×, and 1.4× actual time. The timer used either a numerical (*n* = 16) or non‐numerical cue (*n* = 17). Each individual was exposed to the same type of timer throughout the duration of the experiment. The numerical timer flashed when 10 s elapsed (0, 10, 20, 30, etc.), starting either at 120 and counting down or at 0 and counting up. The non‐numerical timer worked in a similar manner, but an image of a flower flashed on the screen every 10 s.

Time manipulation conditions (*n* = 5 or 7 per individual) were presented in different orders to control for the effect of trial order. Time conditions were not completely randomized, but either increased or decreased one step between trials in an effort to prevent participants from detecting the manipulation.

To minimize the ability of participants to keep track of time during breath holding, each person was asked to read a passage during breath holds. Following each breath hold, participants were asked to answer a multiple‐choice question to verify that they did read the passage. Trials with incorrect answers were excluded from analysis.

### 2.3 Procedure

For each participant, we first measured a baseline maximum breath‐hold duration in the absence of a timer. Participants pressed a button at the beginning of the breath hold, when they began to feel discomfort, and at the end of the breath hold. We then instructed them to close their eyes and indicate their estimate of a 30 s duration (“baseline” time perception) via button presses.

During experimental trials, participants were asked to hold their breath while watching a timer. Again, they pressed the button at the beginning of the breath hold, when they began to feel discomfort, and at the end of the breath hold. The experimenter was blind to the time manipulation condition. To measure the perception of time following each breath hold, participants were instructed to close their eyes and estimate a 30 s duration via button presses.

At the end of the experiment, participants were asked if they noticed anything different about the experimental trials in order to determine whether or not they were able to tell that time was being manipulated.

### 2.4 Analysis

Data collected with LabChart were analyzed to determine the onset of the physiological struggle phase (e.g., see Figure 1), total breath‐hold duration, and perception of time after each apnea trial. The experimenter performing the quantification was blind to the time manipulation condition. Mixed model regressions were used to test whether time manipulation affected the perception of time, overall breath‐hold duration, duration of the easy‐going phase, and duration of the struggle phase. Fixed factors in each model were the time manipulation condition (0.6× to 1.4×, as a numerical indicator), trial number (1–5 or 1–7, as a numerical indicator), and all interaction effects. We included the trial number in the model to control for the fact that apnea duration increases with repeated trials (Schagatay et al., 2000). We also included a random effect of individual. Models were estimated using R (version 3.6.0) and the nlme package (Pinheiro J, Bates D, DebRoy S, Sarkar D, version 3.1‐140). A *p*‐value less than .05 was used to determine significance. All regressions presented in this report are shown as mean ± *SE*.

No difference was found between the results from the numerical and non‐numerical timekeepers, so the data were grouped together. Data from individuals who were aware of the time manipulation (*n* = 7) were excluded.

## 3 RESULTS

### 3.1 The skewed timer affects the perception of time

**Figure 2:**
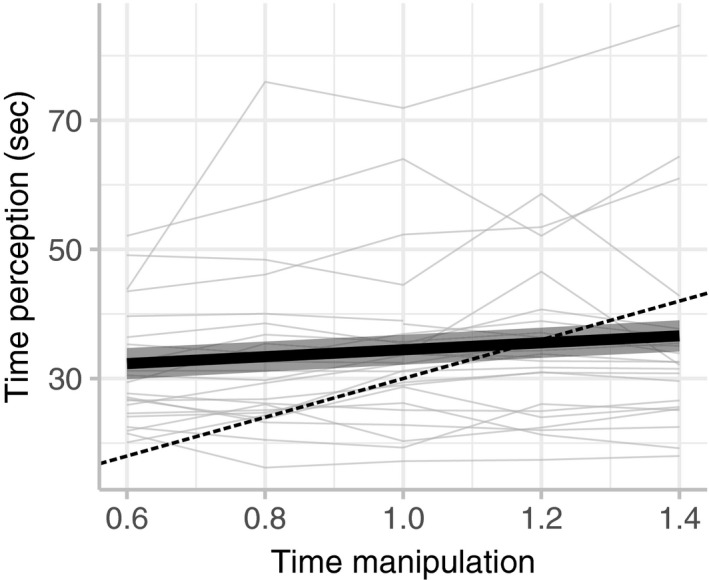
Time manipulation affects the perception of time. Each grey line represents data from one individual (*n* = 11 females, 14 males, 1 unspecified). The significant (*p* = .002) fitted regression line (±*SE*) from the mixed model regression is shown with a thick black line. A dotted black line indicates a 1:1 relationship between time manipulation and time perception

Participants estimated 30 s to be significantly longer or shorter after viewing the skewed timer, based on the timer they had watched during the trial (Figure 2). When time was manipulated to appear to pass faster than actual time, the perception of 30 s was significantly less than when the time was manipulated to pass slower than actual time (*p* = .002; Table 1). Trial number did not significantly affect the perception of time (*p* = .118; Table 1).

**Table 1:** Statistical results for all regressions

	*F*	*df*	*p*
***Perception***			
Trial number	2.47	1,143	.118
**Time manipulation**	**10.10**	**1,143**	**.002**
***Total duration***			
**Trial number**	**17.54**	**1,143**	**<.001**
**Time manipulation**	**11.03**	**1,143**	**.001**
***Easy‐going phase***			
**Trial number**	**6.72**	**1,143**	**.011**
**Time manipulation**	**4.23**	**1,143**	**.042**
***Struggle phase***			
Trial number	3.00	1,143	.085
Time manipulation	1.75	1,143	.187

Note

Bold text indicates significant effects. Mixed model regression results with a random effect for individual.

### 3.2 Time manipulation affects duration of the overall breath hold and duration of the easy‐going phase

**Figure 3:**
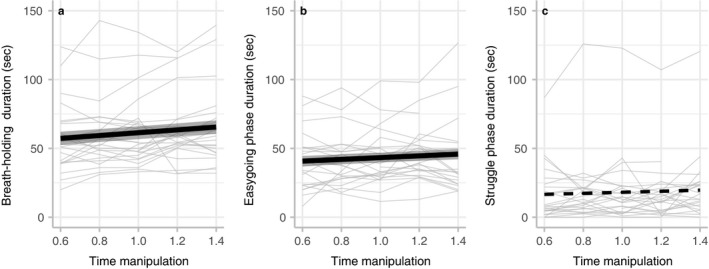
Time manipulation affects the overall breath‐hold duration and the duration of the easy‐going phase, but not the duration of the struggle phase. Each line represents data from one individual (*n* = 11 females, 14 males, 1 unspecified). Significant fitted regression lines (*p* = .001 and *p* = .042 for a and b, respectively) are shown with a thick black line (±*SE*). A non‐significant regression line (*p* = .187) is shown with a thick dashed line (c)

Time manipulation significantly affected breath‐hold duration (*p* = .001, Table 1). Participants held their breath longer when the time was manipulated to be slower (Figure 3a). Breath‐hold durations during experiments were almost always longer than the baseline breath‐hold duration (1.41 ± 0.47 times longer, mean ± *SD*).

The duration of the easy‐going phase increased when time was manipulated to be slower (*p* = .042; Figure 3b). In contrast, the duration of the “struggle” phase was not significantly impacted by time manipulation (*p* = .187; Figure 3c).

## 4 DISCUSSION

This study examined how a psychological factor, the perception of time, impacts the physiological response to apnea. We found that viewing a skewed timer acutely altered participants’ perception of time, even when they were no longer viewing the timer (Figure 2). Overall breath‐hold duration was significantly affected by the time manipulation. When time was manipulated to go slower, the duration increased (Figure 3a). Contrary to our initial hypothesis, the duration of the easy‐going phase, but not the struggle phase, was significantly affected by the time manipulation (Figure 3b–c).

The local task‐centric theory of time perception states that time is perceived relative to an external stimulus (Allman, Teki, Griffiths, & Meck, 2013; Tomassini, Vercillo, Torricelli, & Morrone, 2018). Consistent with this, we found that time perception was skewed following exposure to a distorted timer (Figure 2). This effect is probably not due to the apnea itself. Previous studies have shown that subjective estimates of time (in the range of seconds) may be affected by changes in heart rate (Meissner & Wittmann, 2011), including a reduction in heart rate during breath holding (Di Rienzo, Hoyek, Collet, & Guillot, 2014; Jamin et al., 2004). However, we measured changes in time estimation after participants had already resumed breathing, when heart rate is known to return rapidly to baseline levels (Andersson & Schagatay, 1998). In addition, while time perception can be affected by attention and arousal (Schwarz, Winkler, & Sedlmeier, 2012), which increases following apnea, it is not clear why greater arousal would cause both increases and decreases in time estimation, as we observed. Therefore, the change in time perception is most likely related to the skewed timer and not to the apnea itself.

Because large changes in the speed of the timer can be quite noticeable, we increased or decreased the timer speed by ~15%–30% between trials, which was the range that we found to be imperceptible to most people. Some individuals did notice changes in the timer across trials (*n* = 7 of 33). Their breath‐hold durations were not affected by the time manipulation, and were excluded from the main data set.

Mental state is known to influence breath holding (Bain, Drvis, Dujic, MacLeod, & Ainslie, 2018), particularly in the context of meditation (Bhargava, Gogate, & Mascarenhas, 1988; Laurino et al., 2012). In addition, performing a cognitive task can increase breath‐hold duration (Alpher et al., 1986). Here we show that time manipulation causes both increases and decreases in overall breath‐holding duration. Participants who estimated time to be moving slower also held their breath for more time (Figure 3a), consistent with the idea that unconscious psychological factors and cognitive processes can significantly influence physiological processes.

We expected that manipulation of time perception, a psychological factor, would affect the struggle phase duration, since prior work has shown that the length of the struggle phase depends on psychological factors such as intrinsic motivation and willingness to withstand discomfort (Godfrey & Campbell, 1969; Sütterlin et al., 2013; Thompson‐Lake et al., 2017). However, we did not find a significant change in the duration of the struggle phase due to time manipulation (Figure 3c), perhaps because the participants in this study had little experience with breath holding and a low tolerance for discomfort. Whether manipulation of time perception can modulate the duration of the struggle phase in trained apneic divers remains to be determined, although apnea experience, over the long term, is known to increase both easy‐going and struggle phases (Ferretti, 2001).

Contrary to our hypothesis, we found that time manipulation significantly affected the duration of the easy‐going phase (Figure 3b). When time was manipulated to be moving slower, the onset of involuntary breathing movements (the physiological breaking point) was delayed, and the length of the easy‐going phase increased. A previous study showed that apnea training over 2 weeks can prolong the easy‐going phase (Schagatay et al., 2000), but such changes have not been reported previously over such a short time scale. Surprisingly, the delay in the physiological breaking point was observed even in individuals with little or no prior apneic experience. Taken together, our results suggest that higher cognitive processes, like time estimation, can influence basal metabolic processes, such as respiratory drive, on a rapid time scale.

In this study, involuntary breathing movements were used as a metric for the onset of the physiological breaking point. While we found differences in the onset of the physiological response, further study is needed to better understand the mechanisms that underlie this change. The onset of involuntary breathing movements is thought to depend primarily on the alveolar pressures of CO_2_, and to a lesser extent, of O_2_, in the body (Lin et al., 1974), but the duration of the easy‐going phase can be increased by apnea experience (Ferretti, 2001). To begin to identify the mechanisms underlying the prolonged easy‐going phase in our study, future work will need to measure arterial blood gases and changes in heart rate. Heart rate tends to decrease during apnea (Schagatay, Andersson, & Nielsen, 2007), and it is possible that heart rates also respond to our time manipulation. If heart rate is lower when time is perceived to be slower, this would tend to reduce the demand for oxygen, which might prolong the easy‐going phase (Lin et al., 1974). Additionally, while we observed a robust and statistically significant effect of time manipulation on apnea duration (Figure 3), there was substantial variation among and within individuals, perhaps in part because we did not control the size of the inspiration before each breath hold. Future studies could also manipulate the levels of CO_2_ and O_2_ in the inspiration before the breath hold to examine the role of the blood gases in this psychological effect. By measuring arterial blood gases throughout a breath hold and measuring heart rate while controlling for different alveolar gas compositions, future studies may identify mechanisms underlying the psychological influence, described here, on the body's response to breath holding.

## CONFLICT OF INTEREST

We have no conflicts of interest.
