# Supplementary material for: Manipulating the perception of time affects voluntary breath‐holding duration
Source: Physiol Rep. 2019 Dec 12;7(23):e14309. doi: 10.14814/phy2.14309 (PMC6908740; doi:10.14814/phy2.14309)
Supplement: Supplementary file 1 [file PHY2-7-e14309-s001.docx]

# Supplemental Materials

“VigranData.dat”:

Comma separated value file containing the complete data set used for analysis.

“AnalyzeVigranData.pdf”:

Statistical output from R containing all statistical tests and figures. Also contains documentation of the columns in the data file.

“AnalyzeVigranData.Rmd”:

R notebook containing code to run all statistical tests and generate the figures.
