# Supplementary material for: Manipulating the perception of time affects voluntary breath‐holding duration
Source: Physiol Rep. 2019 Dec 12;7(23):e14309. doi: 10.14814/phy2.14309 (PMC6908740; doi:10.14814/phy2.14309)
Supplement: Supplementary file 2 [file PHY2-7-e14309-s002.pdf]

Manipulating the perception of time affects voluntary breath holding duration

Vigran, Kapral, Tytell, Kao

```r
library(tidyverse)
```

```
## -- Attaching packages --------------------------------------------------------------------- tidyv
## v ggplot2 3.2.1     v purrr   0.3.2
## v tibble  2.1.3     v dplyr   0.8.3
## v tidyr   1.0.0     v stringr 1.4.0
## v readr   1.3.1     v forcats 0.4.0
## -- Conflicts ------------------------------------------------------------------------ tidyverse_c
## x dplyr::filter() masks stats::filter()
## x dplyr::lag()    masks stats::lag()
```

```r
library(nlme)
```

```
##
## Attaching package: 'nlme'
## The following object is masked from 'package:dplyr':
##
##     collapse
```

```r
library(ggbeeswarm)
library(emmeans)
```

```
## Welcome to emmeans.
## NOTE -- Important change from versions <= 1.41:
##     Indicator predictors are now treated as 2-level factors by default.
##     To revert to old behavior, use emm_options(cov.keep = character(0))
```

```r
library(qqplotr)
```

```
##
## Attaching package: 'qqplotr'
## The following objects are masked from 'package:ggplot2':
##
##     stat_qq_line, StatQqLine
```

```r
library(cowplot)
```

```
##
## ********************************************************
## Note: As of version 1.0.0, cowplot does not change the
##   default ggplot2 theme anymore. To recover the previous
##   behavior, execute:
##   theme_set(theme_cowplot())
## ********************************************************
```

```r
library(car)
```

```
## Loading required package: carData
```

```
##
## Attaching package: 'car'
```

```
## The following object is masked from 'package:dplyr':
##
##     recode
```

```
## The following object is masked from 'package:purrr':
##
##     some
```

```r
library(knitr)
library(kableExtra)
```

```
##
## Attaching package: 'kableExtra'
```

```
## The following object is masked from 'package:dplyr':
##
##     group_rows
```

```r
R.Version()$version.string
```

```
## [1] "R version 3.6.1 (2019-07-05)"
```

```r
citation("nlme")
```

```
##
## Pinheiro J, Bates D, DebRoy S, Sarkar D, R Core Team (2019).
## _nlme: Linear and Nonlinear Mixed Effects Models_. R package
## version 3.1-141, <URL: https://CRAN.R-project.org/package=nlme>.
##
## A BibTeX entry for LaTeX users is
##
##   @Manual{,
##     title = {{nlme}: Linear and Nonlinear Mixed Effects Models},
##     author = {Jose Pinheiro and Douglas Bates and Saikat DebRoy and Deepayan Sarkar and {R Core Team
##     year = {2019},
##     note = {R package version 3.1-141},
##     url = {https://CRAN.R-project.org/package=nlme},
##   }
```

Theme for plots for the paper

```r
papertheme <- theme_bw() + theme(axis.line = element_line(color="gray"),
                                 axis.ticks = element_line(color="gray"),
                                 panel.border = element_blank(),
                                 strip.background = element_blank(),
                                 legend.title = element_blank())
# experience.colors <- c("#3779c5", "#c3762b")     # Tufts blue and brown
# experience.colors <- c("#8db600", "#007fff")     # green and blue
# experience.colors <- c("#848482", "#848482")     # gray and gray
# experience.colors <- c("#007fff", "#177245")     # blue and dark green
# experience.colors <- c("#ef1414", "#035EE8")     # red and blue
experience.colors <- c("#000000", "#848484")       # black and gray
```

```
gray = "#b0b0b0"
aware.color <- "#888882"
```

Read in the data

Load the data exported from JMP.

```
apneadata <- read.csv('VigranData.dat')
```

Lots of the column names are converted weirdly, so change them over to something easier to deal with in R.

```
apneadata %>%
  transmute(individual=factor(Individual),
            age=Age,
            gender=Gender,
            height=Height..inches.,
            weight=Weight..lbs.,
            timer.type=Type.of.Timer,
            awareness=Awareness,
            distraction=Distraction,
            time.manipulation=Time.Manipulation,
            trial.num=Time,
            time.perception.sec=Perception.of.Time.Post.Trial,
            apnea.duration.sec=Apnea.Duration,
            breakpoint.physiol.sec=Physiological.Breaking.Point..Actual.,
            breakpoint.psych.sec=Psychological.Breaking.Point..Actual.) ->
  apneadata
```

Here's the data:

```
str(apneadata)
```

```
## 'data.frame':    248 obs. of  14 variables:
##  $ individual            : Factor w/ 33 levels "15","1036","1067",..: 23 23 23 23 23 23 1 1 1 1 ...
##  $ age                   : int  20 20 20 20 20 20 22 22 22 22 ...
##  $ gender                : Factor w/ 3 levels "","Female","Male": 2 2 2 2 2 2 2 2 2 2 ...
##  $ height                : int  67 67 67 67 67 67 69 69 69 69 ...
##  $ weight                : int  148 148 148 148 148 148 157 157 157 157 ...
##  $ timer.type            : Factor w/ 2 levels "Non-numerical",..: 2 2 2 2 2 2 1 1 1 1 ...
##  $ awareness             : Factor w/ 2 levels "Aware","Unaware": 1 1 1 1 1 1 2 2 2 2 ...
##  $ distraction           : Factor w/ 3 levels "","No","Yes": 1 3 3 3 3 3 1 2 3 3 ...
##  $ time.manipulation     : num  NA 1.4 1.2 1 0.8 0.6 NA 0.6 0.8 1 ...
##  $ trial.num             : int  0 1 2 3 4 5 0 1 2 3 ...
##  $ time.perception.sec   : num  44.5 47.5 32.9 31 34.4 28 32.4 32.4 36.8 35.7 ...
##  $ apnea.duration.sec    : num  73 90 98 91 114 ...
##  $ breakpoint.physiol.sec: num  67 87 49 72 98 81 16 16 21 18 ...
##  $ breakpoint.psych.sec  : num  39 48 94 52 42 83 16 16 20 18 ...
```

Create a new variable called `baseline` that indicates which trials are baseline. They're just trial zero, but this makes it easier.

```
apneadata <- apneadata %>%
  mutate(baseline = trial.num == 0)
```

Set the `time.manipulation` variable to be NA for the baseline case.

```r
apneadata <- apneadata %>%
  mutate(time.manipulation = if_else(baseline, NA_real_, time.manipulation))
```

Deal with missing values for the physiological breakpoint. If no IBMs were detected, then the breakpoint is NA. For simplicity, in this case, we set it equal to the total apnea duration.

```r
apneadata <- apneadata %>%
  mutate(breakpoint.physiol.sec = if_else(is.na(breakpoint.physiol.sec), apnea.duration.sec, breakpoint
```

Get rid of some weird values where the time perception variable was negative.

```r
apneadata <- apneadata %>%
  filter(time.perception.sec > 0)
```

Check the number of trials for each time manipulation condition.

```r
xtabs(~factor(time.manipulation), data=apneadata)
```

```
## factor(time.manipulation)
## 0.6 0.8   1 1.2 1.4
##  32  52  44  52  34
```

```r
apneadata %>%
  group_by(awareness, gender, individual) %>%
  tally() %>% tally()
```

```
## # A tibble: 5 x 3
## # Groups:   awareness [2]
##   awareness gender     n
##   <fct>     <fct>  <int>
## 1 Aware     Female     4
## 2 Aware     Male       3
## 3 Unaware   ""         1
## 4 Unaware   Female    11
## 5 Unaware   Male      14
```

```r
apneadata %>%
  group_by(individual) %>%
  summarize(age = mean(age, na.rm=TRUE)) %>%
  summarize(age.mn = mean(age, na.rm=TRUE),
            age.sd = sd(age, na.rm=TRUE))
```

```
## # A tibble: 1 x 2
##   age.mn age.sd
##    <dbl>  <dbl>
## 1   19.6   1.33
```

Here are the individual baseline apnea durations. Colored by gender, but that doesn't seem to matter.

```r
apneadata %>%
  filter(baseline) %>%
  ggplot(aes(y=reorder(individual, apnea.duration.sec), x=apnea.duration.sec,
             color=gender, shape=gender)) +
  geom_point() +
  labs(x='Apnea Duration (s)', y="Individual") +
  theme(axis.text.y = element_blank())
```

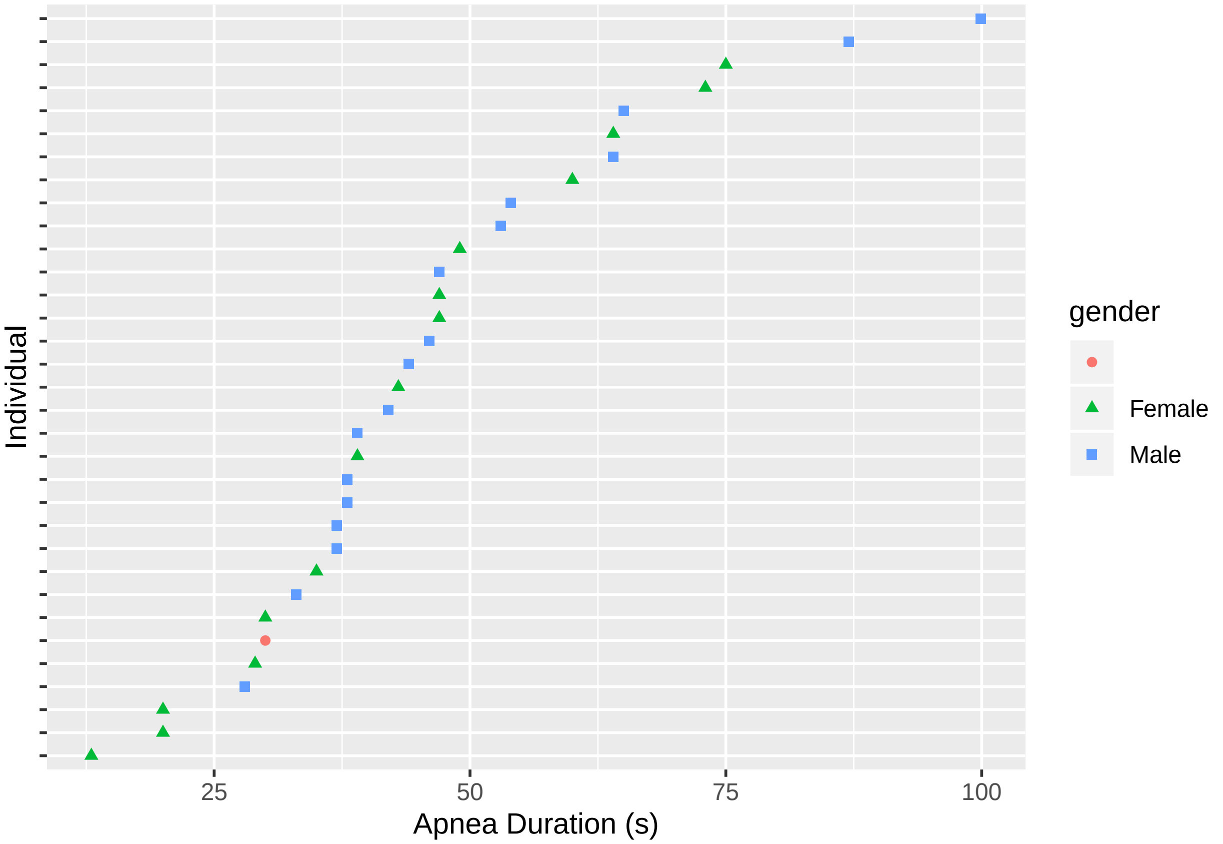

Remove the baseline trials, since we're not normalizing.

```
apneadata %>%
  filter(!baseline) %>%
  mutate(easygoing.sec = breakpoint.physiol.sec,
         struggle.sec = apnea.duration.sec - easygoing.sec) ->
  apneatrials
```

Show the new data frame.

```
str(apneatrials)
```

```
## 'data.frame':    214 obs. of  17 variables:
##  $ individual            : Factor w/ 33 levels "15","1036","1067",..: 23 23 23 23 23 1 1 1 1 1 ...
##  $ age                   : int  20 20 20 20 20 22 22 22 22 22 ...
##  $ gender                : Factor w/ 3 levels "","Female","Male": 2 2 2 2 2 2 2 2 2 2 ...
##  $ height                : int  67 67 67 67 67 69 69 69 69 69 ...
##  $ weight                : int  148 148 148 148 148 157 157 157 157 157 ...
##  $ timer.type            : Factor w/ 2 levels "Non-numerical",..: 2 2 2 2 2 1 1 1 1 1 ...
##  $ awareness             : Factor w/ 2 levels "Aware","Unaware": 1 1 1 1 1 2 2 2 2 2 ...
##  $ distraction           : Factor w/ 3 levels "","No","Yes": 3 3 3 3 3 2 3 3 3 3 ...
##  $ time.manipulation     : num  1.4 1.2 1 0.8 0.6 0.6 0.8 1 1.2 1.4 ...
##  $ trial.num             : int  1 2 3 4 5 1 2 3 4 5 ...
##  $ time.perception.sec   : num  47.5 32.9 31 34.4 28 32.4 36.8 35.7 37.1 33.9 ...
##  $ apnea.duration.sec    : num  90 98 91 114 108 ...
##  $ breakpoint.physiol.sec: num  87 49 72 98 81 16 21 18 29 23 ...
##  $ breakpoint.psych.sec  : num  48 94 52 42 83 16 20 18 20 22 ...
##  $ baseline              : logi  FALSE FALSE FALSE FALSE FALSE FALSE ...
```

```
##  $ easygoing.sec         : num  87 49 72 98 81 16 21 18 29 23 ...
##  $ struggle.sec          : num  3 49 19 15.5 27.2 ...
```

And set up a subset that contains just the unaware individuals.

```
apneatrials.unaware <- filter(apneatrials, awareness == "Unaware")
```

Check the number of trials for each time manipulation condition, once we exclude the aware people.

```
xtabs(~factor(time.manipulation), data=apneatrials.unaware)
```

```
## factor(time.manipulation)
## 0.6 0.8   1 1.2 1.4
##  26  44  34  41  26
```

Expected effects

We expect that apnea duration will increase over successive trials.

Apnea duration as a function of trial number

Let's look at apnea duration relative to time. Set up our list of stats models.

```
lm.dur <- list()

lm.dur[["base"]] <- lme(apnea.duration.sec ~ trial.num,
                   random = ~1 | individual,
                   method = "ML",
                   data = apneatrials.unaware)
lm.dur[["time"]] <- lme(apnea.duration.sec ~ trial.num + time.manipulation,
                   random = ~1 | individual,
                   method = "ML",
                   data = apneatrials.unaware)
lm.dur[["full"]] <- lme(apnea.duration.sec ~ trial.num * time.manipulation,
                   random = ~1 | individual,
                   method = "ML",
                   data = apneatrials.unaware)
```

Compare them:

```
anova(lm.dur[["base"]], lm.dur[["time"]], lm.dur[["full"]])
```

```
##                   Model df      AIC      BIC    logLik   Test   L.Ratio
## lm.dur[["base"]]     1  4 1378.957 1391.524 -685.4784
## lm.dur[["time"]]     2  5 1370.185 1385.893 -680.0925 1 vs 2 10.771911
## lm.dur[["full"]]     3  6 1370.794 1389.644 -679.3970 2 vs 3  1.390957
##                   p-value
## lm.dur[["base"]]
## lm.dur[["time"]]  0.0010
## lm.dur[["full"]]  0.2382
```

The model with time manipulation and experience is just slightly (but not significantly) better than the one with just time manipulation. And the full model is not any better.

```
summary(lm.dur[["time"]])
```

```
## Linear mixed-effects model fit by maximum likelihood
##  Data: apneatrials.unaware
```

```
##        AIC      BIC    logLik
##   1370.185 1385.893 -680.0925
##
## Random effects:
##  Formula: ~1 | individual
##         (Intercept) Residual
## StdDev:    23.22032 9.800392
##
## Fixed effects: apnea.duration.sec ~ trial.num + time.manipulation
##                      Value Std.Error  DF  t-value p-value
## (Intercept)       45.74303  5.635236 143 8.117323  0.0000
## trial.num          1.39730  0.399685 143 3.496008  0.0006
## time.manipulation 10.27644  3.094936 143 3.320403  0.0011
##  Correlation:
##                   (Intr) trl.nm
## trial.num         -0.167
## time.manipulation -0.497 -0.186
##
## Standardized Within-Group Residuals:
##         Min          Q1         Med          Q3         Max
## -2.98737228 -0.48013029 -0.06233119  0.49152315  3.84475695
##
## Number of Observations: 171
## Number of Groups: 26
```

Here, we're just thinking about the effect of trial number, which is highly significant.

Calculate the predicted marginal means for apnea duration as a function of trial number.

```
lm.dur.trialfit <- as.data.frame(
  emmeans(lm.dur[["time"]], ~trial.num,
          at = list(trial.num = seq(1,7)))
)
```

```
fig.trial <-
  apneatrials.unaware %>%
  ggplot(aes(x = trial.num, y = apnea.duration.sec)) +
  geom_line(aes(group=individual), size=0.5, color=gray) +
  geom_ribbon(data=lm.dur.trialfit, aes(x=trial.num, ymin=emmean-SE, ymax=emmean+SE),
              alpha=0.2, inherit.aes = FALSE) +
  geom_line(data=lm.dur.trialfit, aes(x=trial.num, y=emmean), inherit.aes = FALSE, size=1.5) +
  labs(x="Trial number", y="Apnea duration (sec)") +
  scale_x_continuous(breaks=seq(1,7)) +
  papertheme + theme(panel.grid.minor.x = element_blank())

fig.trial
```

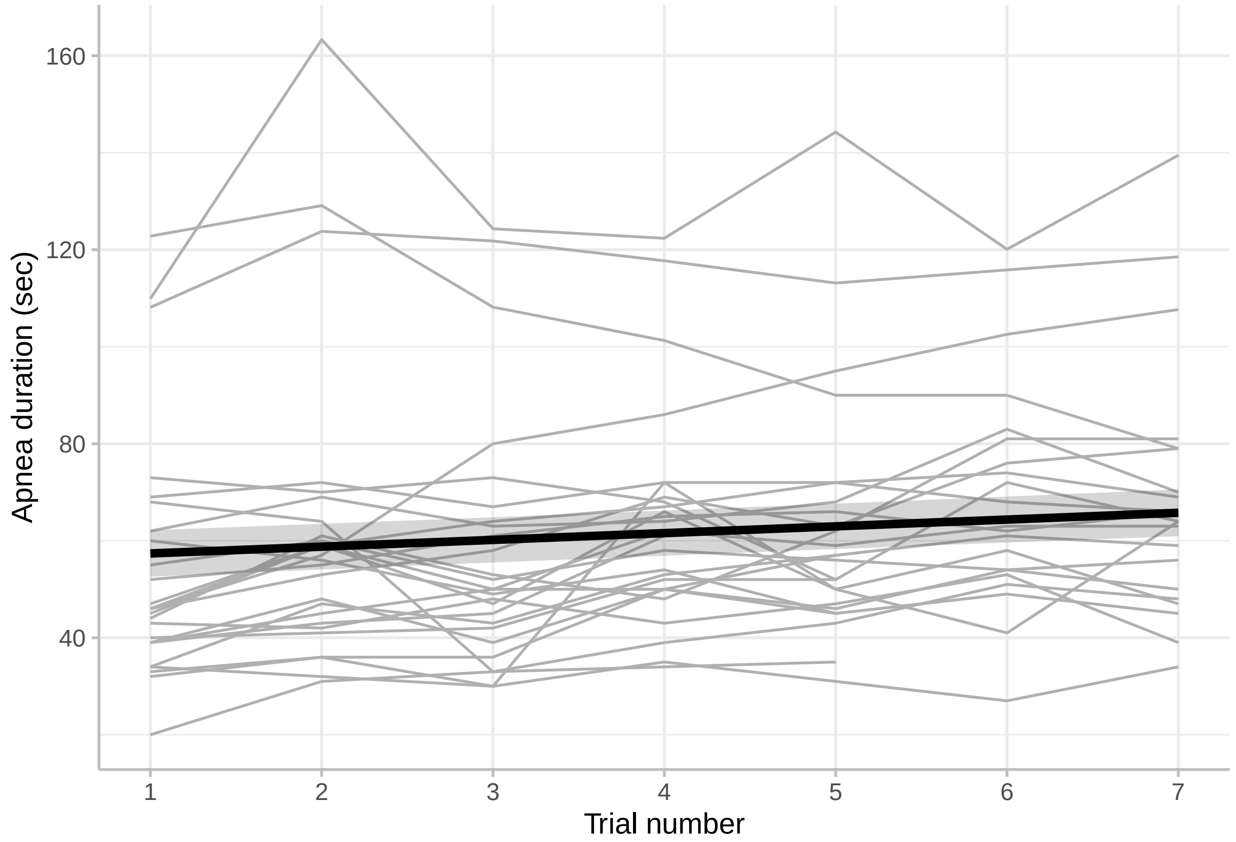

This is the apnea duration as a function of trial number, showing that it generally gets longer in later trials.

```
norm <-
  apneadata %>%
  filter(awareness == "Unaware") %>%
  group_by(individual, baseline) %>%
  summarize(dur.mn = mean(apnea.duration.sec)) %>%
  pivot_wider(names_from = baseline, values_from = dur.mn) %>%
  rename(dur.mn.base = `TRUE`, dur.mn = `FALSE`) %>%
  mutate(dur.mn.norm = dur.mn / dur.mn.base) %>%
  ungroup()

norm
```

```
## # A tibble: 26 x 4
##    individual dur.mn dur.mn.base dur.mn.norm
##    <fct>       <dbl>       <dbl>       <dbl>
##  1 15           30.6          20        1.53
##  2 1036         46.6          30        1.55
##  3 1067         52.6          49        1.07
##  4 1201         54.4          43        1.27
##  5 2057         32.3          30        1.08
##  6 2175         61.4          37        1.66
##  7 2741         46.1          38        1.21
##  8 2809        103.           54        1.91
##  9 3002         82.0          47        1.75
## 10 3138         67.4          47        1.43
## # ... with 16 more rows
```

```
norm %>% ungroup() %>%
  summarize(dur.std.norm = sd(dur.mn.norm, na.rm = TRUE), dur.mn.norm = mean(dur.mn.norm, na.rm=TRUE))
```

```
## # A tibble: 1 x 2
##   dur.std.norm dur.mn.norm
##          <dbl>       <dbl>
## 1        0.471        1.41
```

Time perception

Here's a baseline plot of everyone's time perception against the time manipulation condition.

```
ggplot(apneatrials.unaware, aes(x=time.manipulation, y=time.perception.sec)) +
  geom_line(aes(group=individual))
```

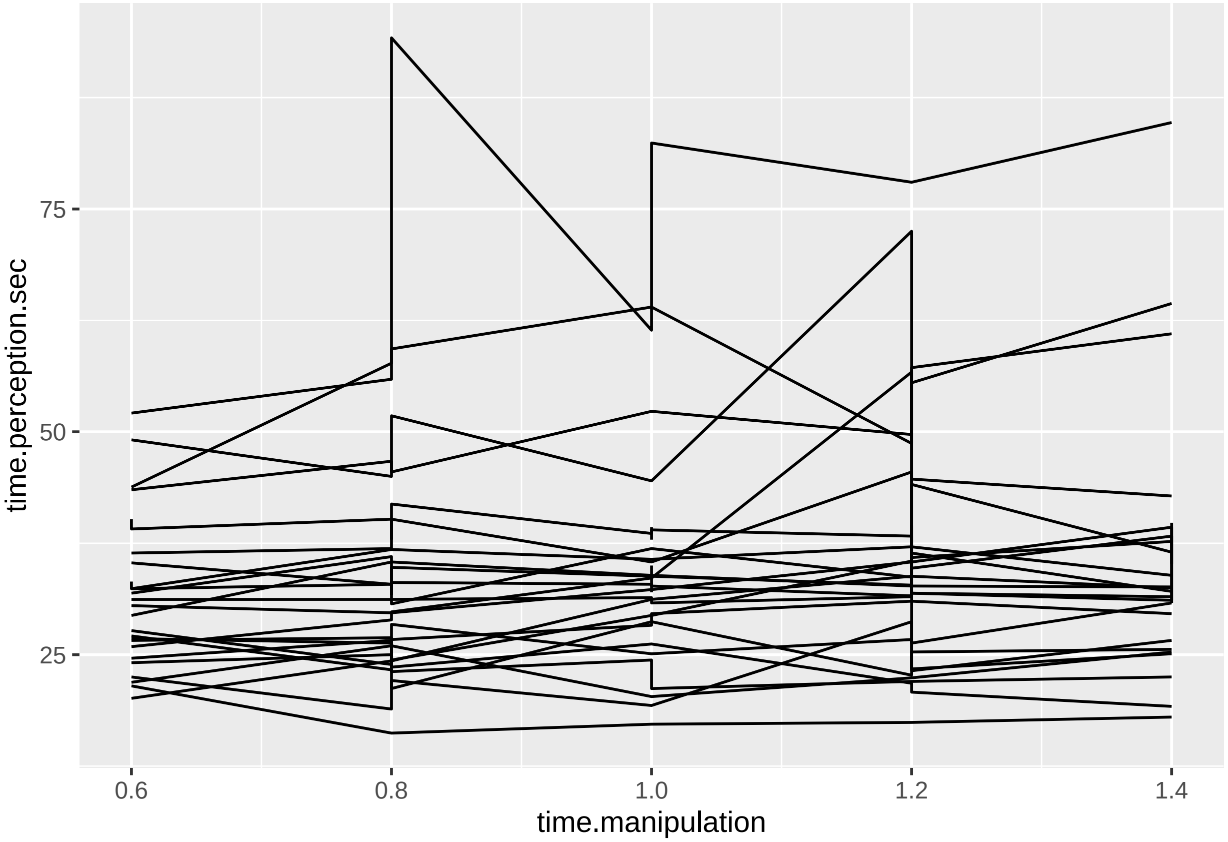

Check a set of statistical models. We'll use AIC to choose the best model across all of the different independent variables we're running, but then we'll run the same model for each of them.

```
lm.perception <- list()
lm.perception[["base"]] <- lme(time.perception.sec ~ 1,
              random = ~1 | individual,
              na.action = na.omit,
              method="ML",
              data = apneatrials.unaware)
lm.perception[["trial"]] <- lme(time.perception.sec ~ trial.num,
              random = ~1 | individual,
              na.action = na.omit,
```

```
              method="ML",
              data = apneatrials.unaware)
lm.perception[["time"]] <- lme(time.perception.sec ~ trial.num + time.manipulation,
              random = ~1 | individual,
              na.action = na.omit,
              method="ML",
              data = apneatrials.unaware)
lm.perception[["full"]] <- lme(time.perception.sec ~ trial.num * time.manipulation,
              random = ~1 | individual,
              na.action = na.omit,
              method="ML",
              data = apneatrials.unaware)
lm.perception[["anova"]] <- lme(time.perception.sec ~ factor(trial.num) + factor(time.manipulation),
                                random = ~1 | individual,
                                na.action = na.omit,
                                method = "ML",
                                data = apneatrials.unaware)
```

```
anova(lm.perception[["base"]], lm.perception[["time"]], lm.perception[["full"]])
```

```
##                           Model df      AIC      BIC    logLik   Test
## lm.perception[["base"]]     1  3 1166.859 1176.284 -580.4297
## lm.perception[["time"]]     2  5 1158.602 1174.310 -574.3011 1 vs 2
## lm.perception[["full"]]     3  6 1160.227 1179.076 -574.1133 2 vs 3
##                             L.Ratio p-value
## lm.perception[["base"]]
## lm.perception[["time"]] 12.257245  0.0022
## lm.perception[["full"]]  0.375576  0.5400
```

Here, the model with time manipulation only is the best one. Combining time and trial number doesn't add anything.

```
anova(lm.perception[["time"]], lm.perception[["anova"]])
```

```
##                            Model df      AIC      BIC    logLik   Test
## lm.perception[["time"]]      1  5 1158.602 1174.310 -574.3011
## lm.perception[["anova"]]     2 13 1172.190 1213.032 -573.0950 1 vs 2
##                             L.Ratio p-value
## lm.perception[["time"]]
## lm.perception[["anova"]] 2.412031  0.9657
```

So, treating the trial number and time manipulation as categorical variables results in a worse model.

```
anova(lm.perception[["base"]], lm.perception[["trial"]])
```

```
##                            Model df      AIC      BIC    logLik   Test
## lm.perception[["base"]]      1  3 1166.859 1176.284 -580.4297
## lm.perception[["trial"]]     2  4 1166.520 1179.086 -579.2599 1 vs 2
##                            L.Ratio p-value
## lm.perception[["base"]]
## lm.perception[["trial"]] 2.33965  0.1261
```

Also trial isn't a significant effect.

```
summary(lm.perception[["time"]])
```

```
## Linear mixed-effects model fit by maximum likelihood
##  Data: apneatrials.unaware
```

```
##        AIC     BIC    logLik
##   1158.602 1174.31 -574.3011
##
## Random effects:
##  Formula: ~1 | individual
##         (Intercept) Residual
## StdDev:    11.49495  5.35686
##
## Fixed effects: time.perception.sec ~ trial.num + time.manipulation
##                       Value Std.Error  DF  t-value p-value
## (Intercept)       28.284364 2.8900847 143 9.786690  0.0000
## trial.num          0.207856 0.2184379 143 0.951557  0.3429
## time.manipulation  5.373655 1.6911605 143 3.177496  0.0018
##  Correlation:
##                   (Intr) trl.nm
## trial.num         -0.178
## time.manipulation -0.530 -0.186
##
## Standardized Within-Group Residuals:
##        Min         Q1        Med         Q3        Max
## -4.5171503 -0.4882917 -0.1025357  0.3553610  4.5743145
##
## Number of Observations: 171
## Number of Groups: 26
```

```
plot(lm.perception[["time"]])
```

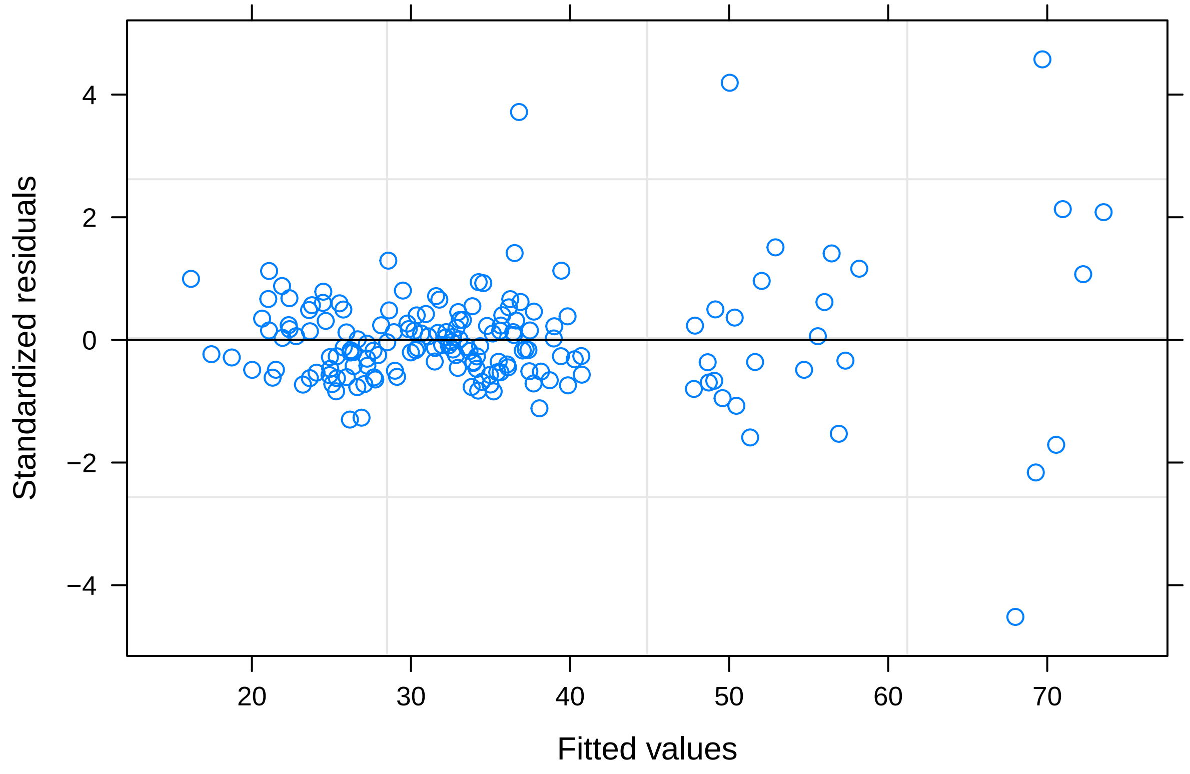

Residuals look OK.

```
ggplot(data.frame(resid=residuals(lm.perception[["time"]])), aes(sample=resid)) +
  stat_qq_line() + stat_qq_band() + stat_qq_point() +
```

```
  labs(x = "Theoretical", y="Sample")
```

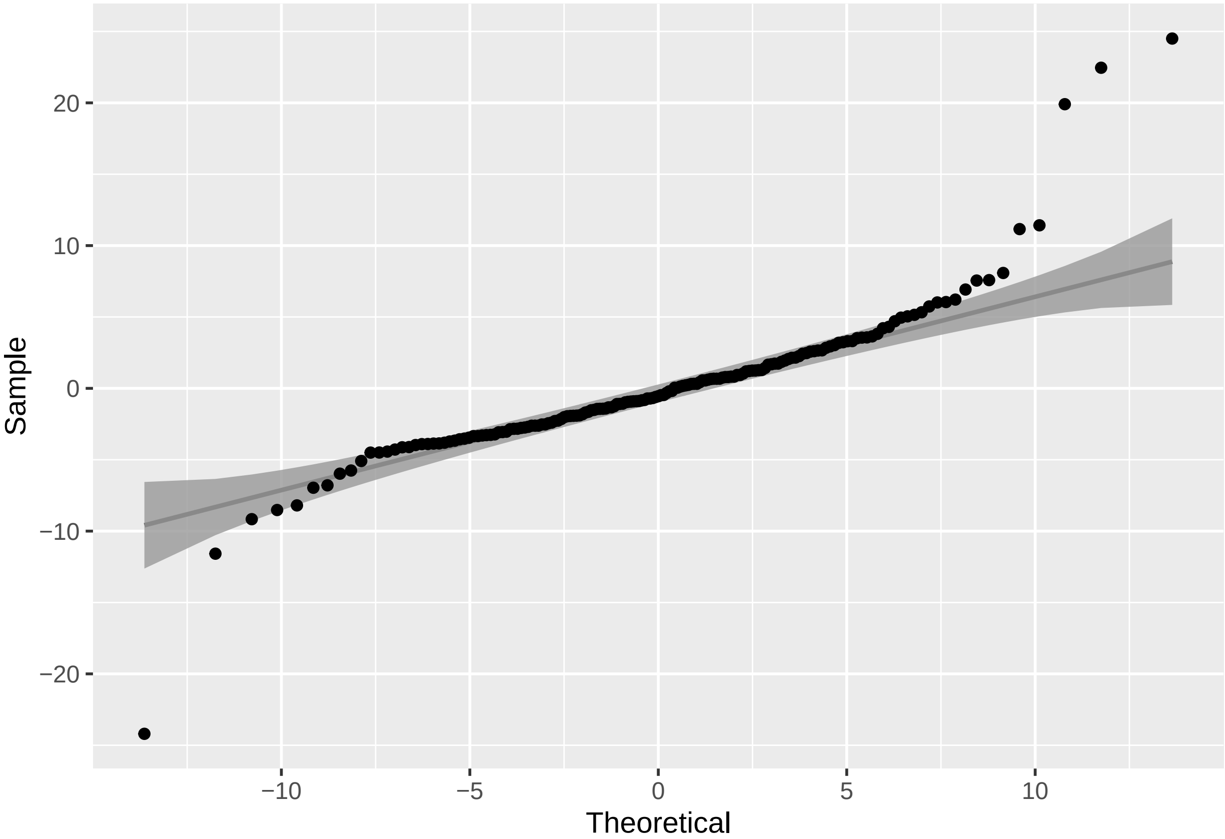

And they're not too far from normal. A bit of positive skew, but I don't think that's going to be a problem.

```
linearHypothesis(lm.perception[["time"]], "time.manipulation = 1")
```

```
## Linear hypothesis test
##
## Hypothesis:
## time.manipulation = 1
##
## Model 1: restricted model
## Model 2: time.perception.sec ~ trial.num + time.manipulation
##
##   Df  Chisq Pr(>Chisq)
## 1
## 2  1 6.8078   0.009076 **
## ---
## Signif. codes:  0 '***' 0.001 '**' 0.01 '*' 0.05 '.' 0.1 ' ' 1
```

If our time manipulation directly affected time perception, we would expect that the slope would be 1. Here, we check that, and find that the slope is significantly less than one.

Generate the predicted marginal means for time manipulation, averaging across the other effects.

```
lm.perception.fit <- as.data.frame(emmeans(lm.perception[["time"]], ~time.manipulation,
                          at = list(time.manipulation = seq(0.6, 1.4, by=0.2))))
```

And generate the plot.

```
fig.perception <-
  apneatrials.unaware %>%
  group_by(individual, time.manipulation) %>%
  summarize(time.perception.sec = mean(time.perception.sec, na.rm=TRUE)) %>%
  ggplot(aes(x = time.manipulation, y = time.perception.sec)) +
  geom_line(aes(group=individual), size=0.5, color=gray) + #, position=position_jitter(width=0.01)) +
  geom_ribbon(data=lm.perception.fit, aes(x=time.manipulation, ymin=emmean-SE, ymax=emmean+SE),
              alpha=0.5, inherit.aes = FALSE) +
  geom_line(data=lm.perception.fit, aes(x=time.manipulation, y=emmean), inherit.aes = FALSE, size=1.5) +
  # geom_line(data=apneatrials.aware, aes(group=individual), color=aware.color, linetype=3, size=0.5) +
  geom_abline(slope=30, intercept=0, color="black", linetype=3, size=1) +
  labs(x="Time manipulation", y="Time perception (sec)") +
  papertheme

fig.perception
```

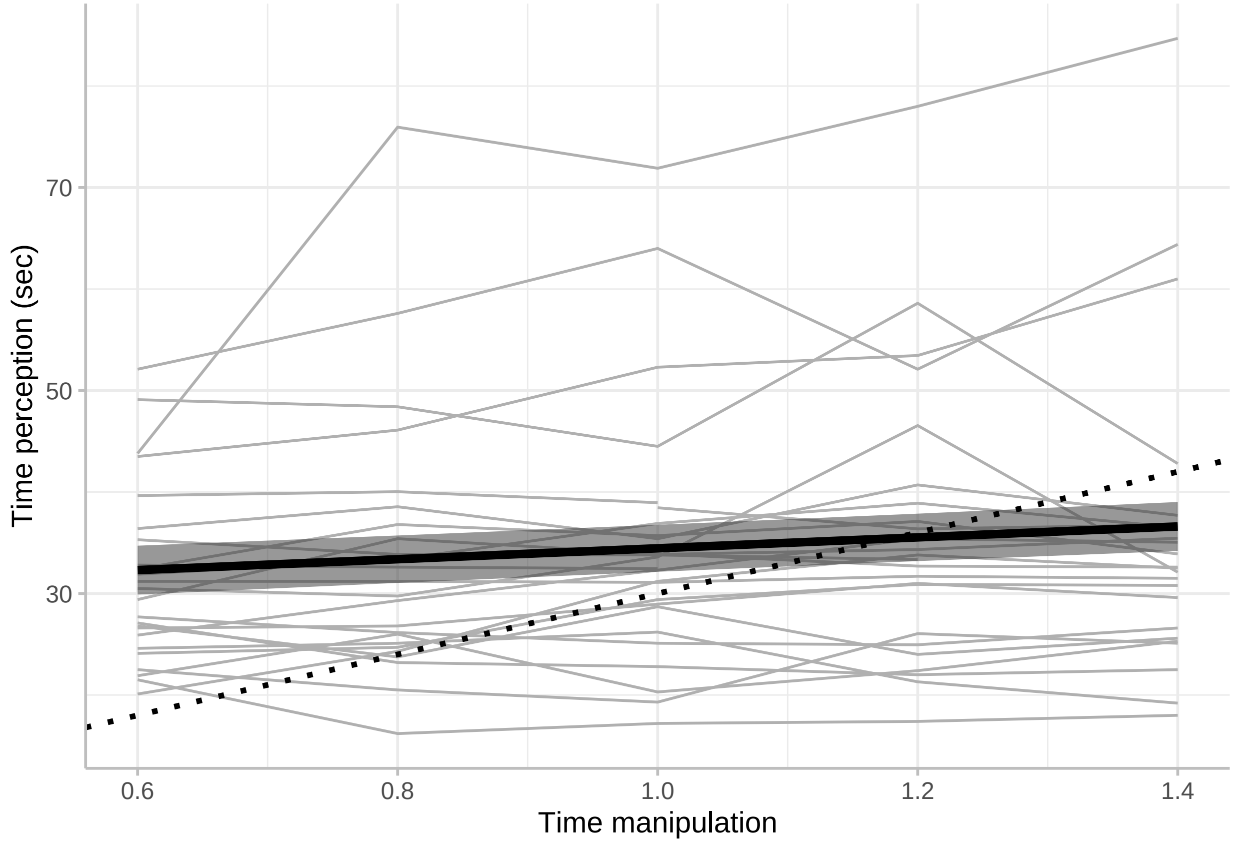


```
filename <- 'Fig-perception.pdf'
ggsave(filename, plot=fig.perception + theme(legend.position = "none"),
       width=3,
       height=2.5, units="in", useDingbats=FALSE)
knitr::include_graphics(filename)
```

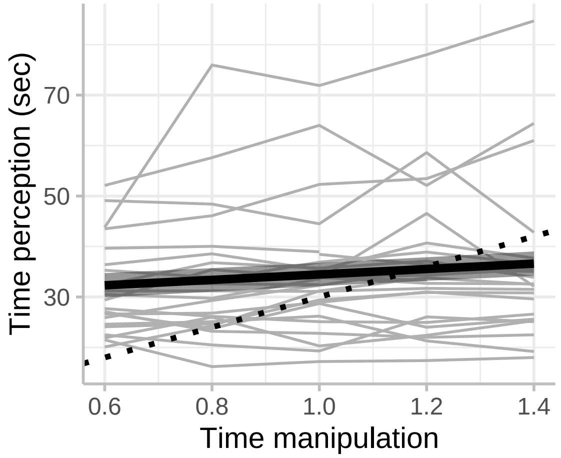

Fig. 4. Time manipulation affects the perception of time. Red solid line, no apnea experience; blue dashed lines, apnea experience. Each line represents one individual. The fitted regression line is shown with a thick black line, and a 1:1 relationship is shown by a dotted black line.

Apnea duration relative to time manipulation

Are apnea durations longer when we manipulate time to be slower?

```
apneatrials %>%
  group_by(individual, time.manipulation) %>%
  summarize(apnea.duration.sec = mean(apnea.duration.sec),
            awareness=awareness[1]) %>%
  arrange(individual, time.manipulation) %>%
  ggplot(aes(x=time.manipulation, y=apnea.duration.sec, color=awareness)) +
  geom_path(aes(group=individual))
```

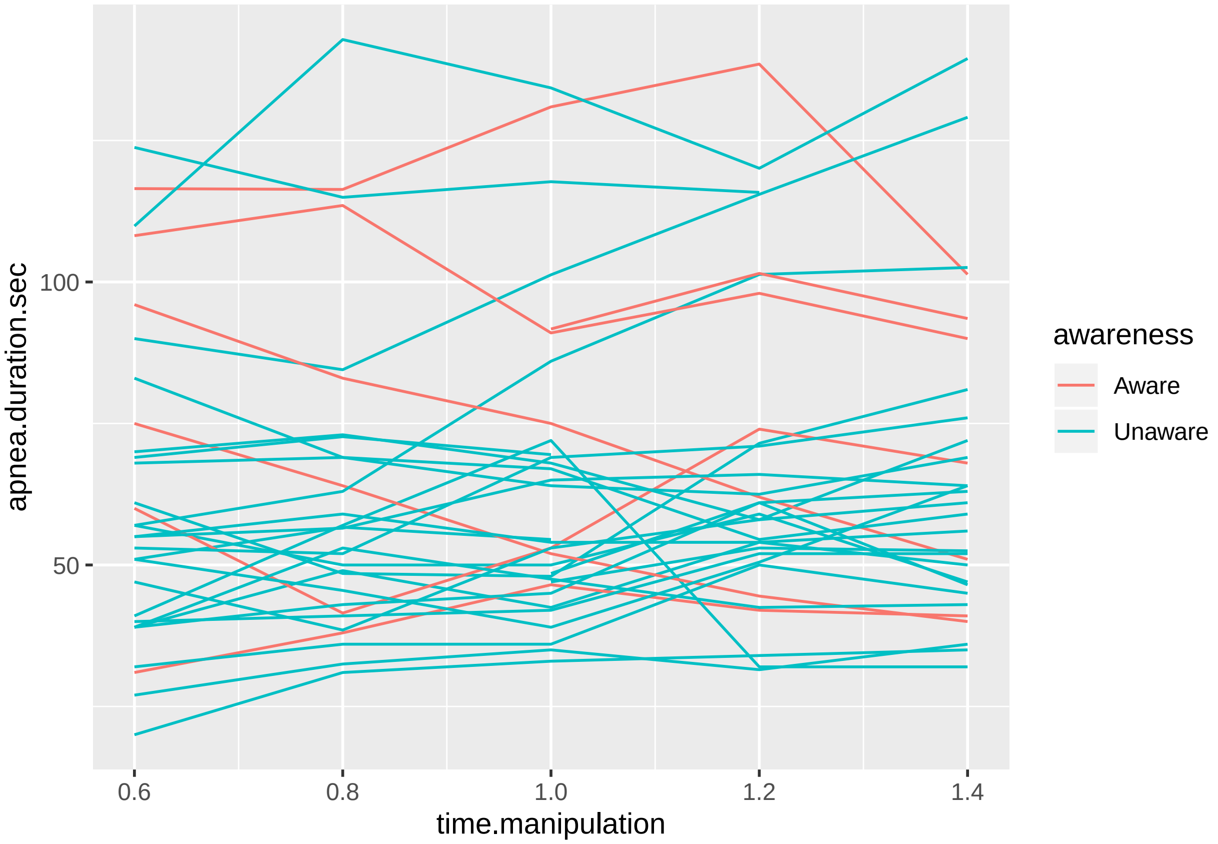

Looks like the are, provided people are unaware. All the downward sloping lines are aware.

Set up our list of stats models.

```
lm.dur <- list()

lm.dur[["base"]] <- lme(apnea.duration.sec ~ trial.num,
                   random = ~1 | individual,
                   method = "ML",
                   data = apneatrials.unaware)
lm.dur[["time"]] <- lme(apnea.duration.sec ~ trial.num + time.manipulation,
                   random = ~1 | individual,
                   method = "ML",
                   data = apneatrials.unaware)
lm.dur[["full"]] <- lme(apnea.duration.sec ~ trial.num * time.manipulation,
                   random = ~1 | individual,
                   method = "ML",
                   data = apneatrials.unaware)
lm.dur[["randslope"]] <- lme(apnea.duration.sec ~ trial.num + time.manipulation,
                        random = ~1 + time.manipulation | individual,
                        method = "ML",
                        data = apneatrials.unaware)
```

Compare them:

```
anova(lm.dur[["base"]], lm.dur[["time"]], lm.dur[["full"]])
```

```
##                   Model df      AIC      BIC    logLik   Test   L.Ratio
## lm.dur[["base"]]      1  4 1378.957 1391.524 -685.4784
```

```
## lm.dur[["time"]]     2  5 1370.185 1385.893 -680.0925 1 vs 2 10.771911
## lm.dur[["full"]]     3  6 1370.794 1389.644 -679.3970 2 vs 3  1.390957
##                  p-value
## lm.dur[["base"]]
## lm.dur[["time"]]  0.0010
## lm.dur[["full"]]  0.2382
```

The model with time manipulation and experience is just slightly (but not significantly) better than the one with just time manipulation. We'll continue using it as our standard model.

```r
anova(lm.dur[["time"]], lm.dur[["randslope"]])
```

```
##                       Model df      AIC      BIC    logLik   Test  L.Ratio
## lm.dur[["time"]]          1  5 1370.185 1385.893 -680.0925
## lm.dur[["randslope"]]     2  7 1358.395 1380.387 -672.1976 1 vs 2 15.78967
##                       p-value
## lm.dur[["time"]]
## lm.dur[["randslope"]]   4e-04
```

```r
summary(lm.dur[["time"]])
```

```
## Linear mixed-effects model fit by maximum likelihood
##  Data: apneatrials.unaware
##        AIC      BIC    logLik
##   1370.185 1385.893 -680.0925
##
## Random effects:
##  Formula: ~1 | individual
##         (Intercept) Residual
## StdDev:    23.22032 9.800392
##
## Fixed effects: apnea.duration.sec ~ trial.num + time.manipulation
##                      Value Std.Error  DF  t-value p-value
## (Intercept)       45.74303  5.635236 143 8.117323  0.0000
## trial.num          1.39730  0.399685 143 3.496008  0.0006
## time.manipulation 10.27644  3.094936 143 3.320403  0.0011
##  Correlation:
##                   (Intr) trl.nm
## trial.num         -0.167
## time.manipulation -0.497 -0.186
##
## Standardized Within-Group Residuals:
##         Min          Q1         Med          Q3         Max
## -2.98737228 -0.48013029 -0.06233119  0.49152315  3.84475695
##
## Number of Observations: 171
## Number of Groups: 26
```

Apnea duration is significantly longer in later trials and when time is manipulated to be longer. Experience tends to affect the duration, but it's not significant. Strangely, it's the unexperienced folks who tend to have longer apneas. But that's because they're normalized - unexperienced people have longer apneas *relative to* their baseline, not necessarily longer overall.

```r
plot(lm.dur[["time"]])
```

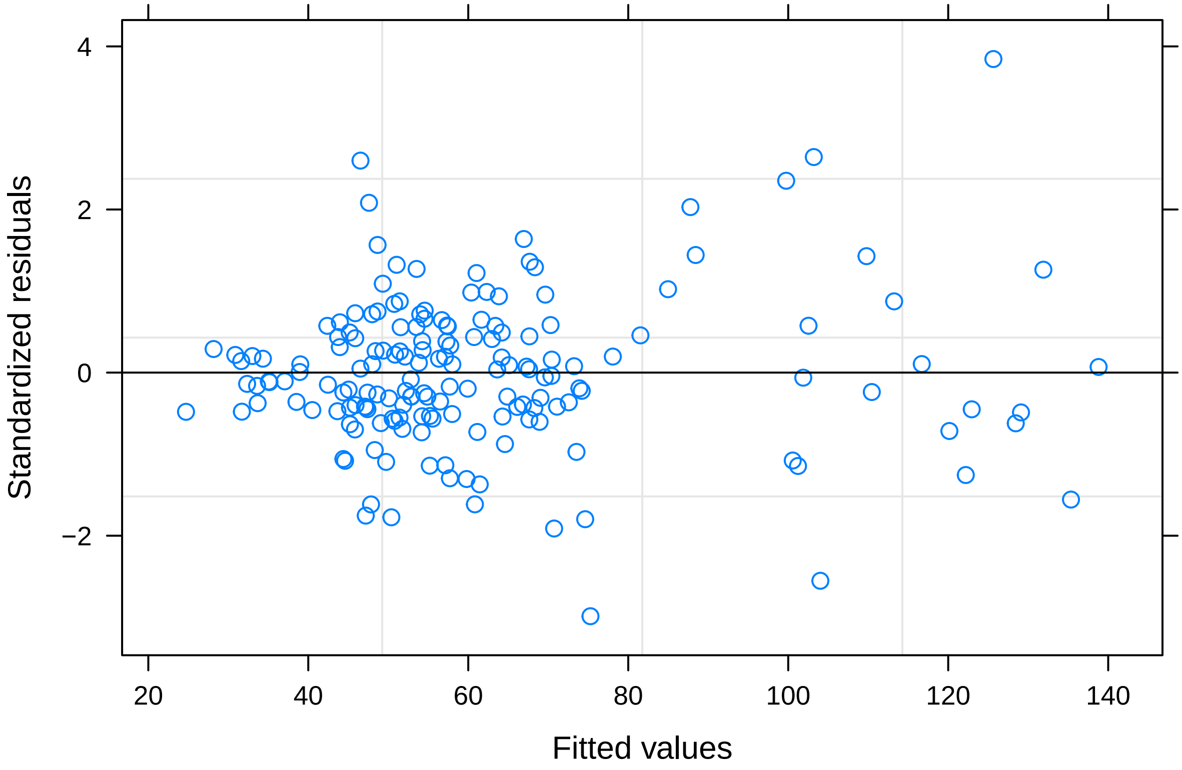


```r
ggplot(data.frame(resid=residuals(lm.dur[["time"]])), aes(sample=resid)) +
  stat_qq_line() + stat_qq_band() + stat_qq_point() +
  labs(x = "Theoretical", y="Sample")
```

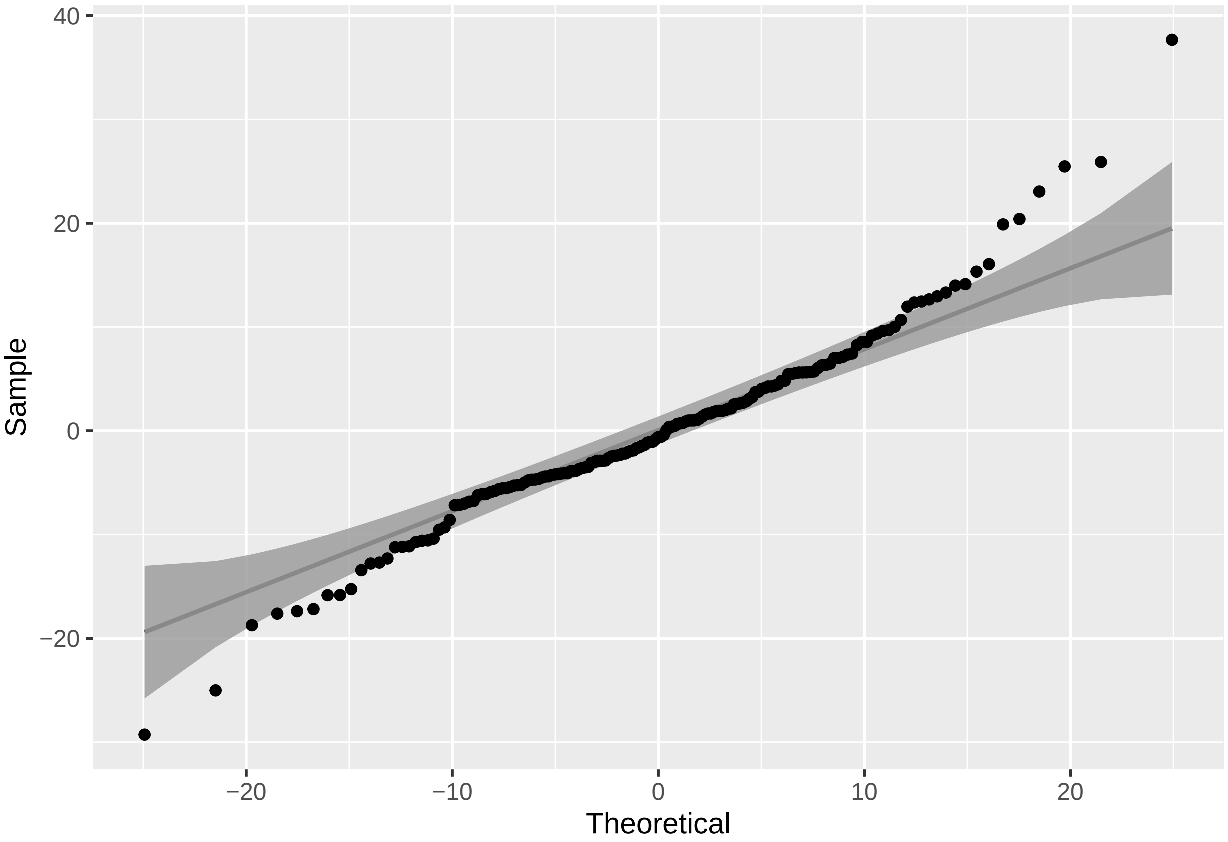

The tails are a bit longer than we'd prefer, but not too bad.

```
summary(lm.dur[["randslope"]])
```

```
## Linear mixed-effects model fit by maximum likelihood
##  Data: apneatrials.unaware
##        AIC      BIC    logLik
##   1358.395 1380.387 -672.1976
##
## Random effects:
##  Formula: ~1 + time.manipulation | individual
##  Structure: General positive-definite, Log-Cholesky parametrization
##                  StdDev    Corr
## (Intercept)       27.088290 (Intr)
## time.manipulation 17.473163 -0.529
## Residual           8.517555
##
## Fixed effects: apnea.duration.sec ~ trial.num + time.manipulation
##                      Value Std.Error  DF  t-value p-value
## (Intercept)       46.35815  6.147588 143 7.540868  0.0000
## trial.num          1.35645  0.458462 143 2.958706  0.0036
## time.manipulation  9.77813  4.458773 143 2.193010  0.0299
##  Correlation:
##                   (Intr) trl.nm
## trial.num         -0.176
## time.manipulation -0.603 -0.150
##
## Standardized Within-Group Residuals:
```

```
##         Min          Q1         Med          Q3         Max
## -2.6770417 -0.4564529 -0.0508728  0.4835831  4.2981496
##
## Number of Observations: 171
## Number of Groups: 26
```

Time manipulation is still significant even if we allow individuals to have their own random slope.

Generate the predicted values for apnea duration as time manipulation changes and as trial number changes.

```
lm.dur.fit <- as.data.frame(
  emmeans(lm.dur[["time"]], ~time.manipulation,
          at = list(time.manipulation = seq(0.6, 1.4, by=0.2))))
```

Show the apnea duration relative to time manipulation. Solid blue lines are with experience; green dashed lines are without. Dotted lines are aware. The red line is a 1:1 relationship.

```
apneatrials.unaware %>%
  group_by(individual, time.manipulation) %>%
  summarize(apnea.duration.sec = mean(apnea.duration.sec, na.rm=TRUE)) %>%
  ggplot(aes(x = time.manipulation, y = apnea.duration.sec)) +
  geom_line(aes(group=individual), size=0.5, color=gray) +
  geom_ribbon(data=lm.dur.fit, aes(x=time.manipulation, ymin=emmean-SE, ymax=emmean+SE),
              alpha=0.2, inherit.aes = FALSE) +
  geom_line(data=lm.dur.fit, aes(x=time.manipulation, y=emmean), inherit.aes = FALSE, size=1.5) +
  geom_abline(slope=1, intercept=0, color="black", linetype=3, size=1) +
  labs(x="Time manipulation", y="Apnea duration (sec)") +
  papertheme
```

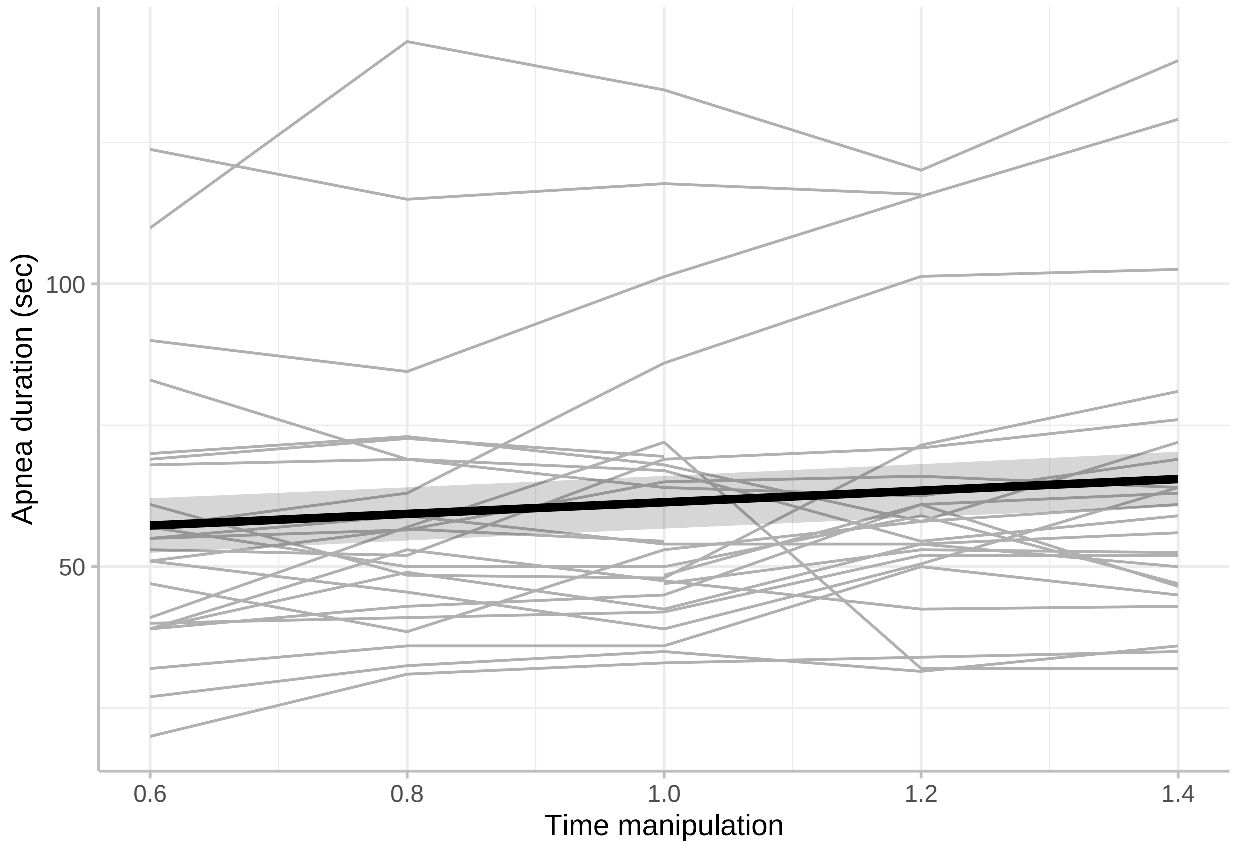

```r
apneatrials %>%
  filter(time.manipulation == 1.0) %>%
  group_by(individual) %>%
  summarize(apnea.duration.1 = mean(apnea.duration.sec)) %>%
  left_join(apneatrials.unaware, by="individual") %>%
  mutate(apnea.duration.norm1 = apnea.duration.sec / apnea.duration.1) ->
  apneatrials.norm1
  # mutate(easygoing.sec = breakpoint.physiol,
  #        struggle.sec = apnea.duration - easygoing.sec,
  #        apnea.duration.norm = apnea.duration / apnea.duration.base,
  #        easygoing.norm = breakpoint.physiol / apnea.duration.base,
  #        struggle.norm = (apnea.duration - breakpoint.physiol) /
  #          apnea.duration.base,
  #        breakpoint.psych.norm = breakpoint.psych / apnea.duration.base,
  #        time.perception.norm = time.perception / time.perception.base) %>%
  # rename(apnea.duration.sec = apnea.duration,
  #        time.perception.sec = time.perception) ->
  # apneadata
```

```r
apneatrials.norm1 %>%
  group_by(individual, time.manipulation) %>%
  summarize(apnea.duration.norm1 = mean(apnea.duration.norm1, na.rm=TRUE)) %>%
  ggplot(aes(x = time.manipulation-1, y = apnea.duration.norm1)) +
  geom_line(aes(group=individual), size=0.5) +
  geom_abline(slope=1.0, intercept=0.0)
```

```
## Warning: Removed 7 rows containing missing values (geom_path).
```

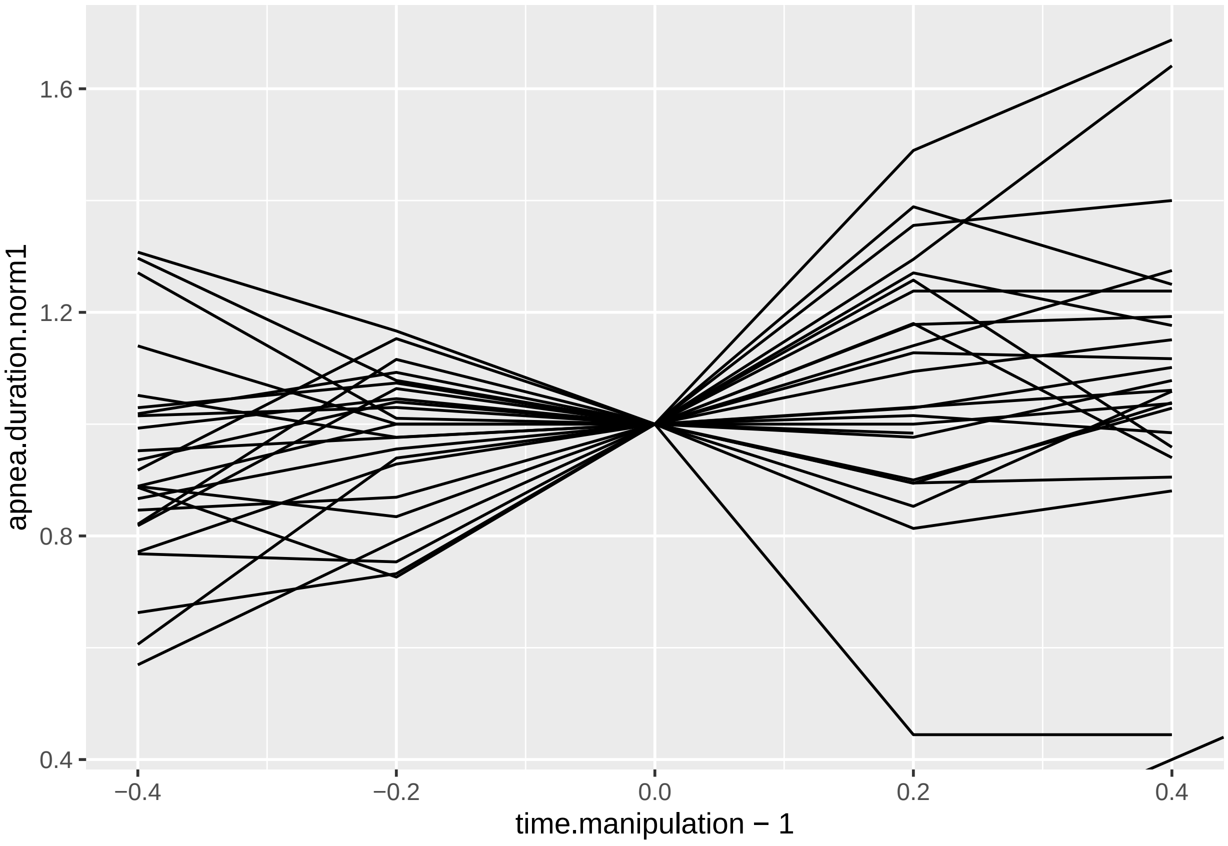

```r
lm.dur[["norm1"]] <- lme(apnea.duration.norm1 ~ trial.num + time.manipulation,
                   random = ~1 | individual,
                   method = "ML",
                   na.action = na.omit,
                   data = apneatrials.norm1)
```

```r
summary(lm.dur[["norm1"]])
```

```
## Linear mixed-effects model fit by maximum likelihood
##  Data: apneatrials.norm1
##         AIC       BIC   logLik
##   -108.6817 -92.97338 59.34085
##
## Random effects:
##  Formula: ~1 | individual
##         (Intercept)  Residual
## StdDev:   0.1055001 0.1536818
##
## Fixed effects: apnea.duration.norm1 ~ trial.num + time.manipulation
##                       Value  Std.Error  DF   t-value p-value
## (Intercept)       0.7378979 0.05485036 143 13.452927   0e+00
## trial.num         0.0231523 0.00623761 143  3.711727   3e-04
## time.manipulation 0.1906636 0.04795493 143  3.975891   1e-04
##  Correlation:
##                   (Intr) trl.nm
## trial.num         -0.273
## time.manipulation -0.792 -0.184
##
## Standardized Within-Group Residuals:
##         Min          Q1         Med          Q3         Max
## -2.46579430 -0.59376941 -0.06416125  0.48985425  3.67507961
##
## Number of Observations: 171
## Number of Groups: 26
```

We could try to compare the slope of the effect to what we'd expect if people responded 1:1 to the change in

```r
linearHypothesis(lm.dur[["norm1"]], "time.manipulation = 1")
```

```
## Linear hypothesis test
##
## Hypothesis:
## time.manipulation = 1
##
## Model 1: restricted model
## Model 2: apnea.duration.norm1 ~ trial.num + time.manipulation
##
##   Df  Chisq Pr(>Chisq)
## 1
## 2  1 289.92  < 2.2e-16 ***
## ---
## Signif. codes:  0 '***' 0.001 '**' 0.01 '*' 0.05 '.' 0.1 ' ' 1
```

The slope of the effect is significantly different from 1.

Easygoing phase

```
easy.sec.mn <- apneatrials.unaware %>%
  group_by(individual, time.manipulation) %>%
  summarize(easygoing.sec = mean(easygoing.sec, na.rm=TRUE),
            apnea.duration.sec = mean(apnea.duration.sec, na.rm=TRUE))

ggplot(easy.sec.mn, aes(x=time.manipulation, y=easygoing.sec)) +
  geom_line(aes(group=individual))
```

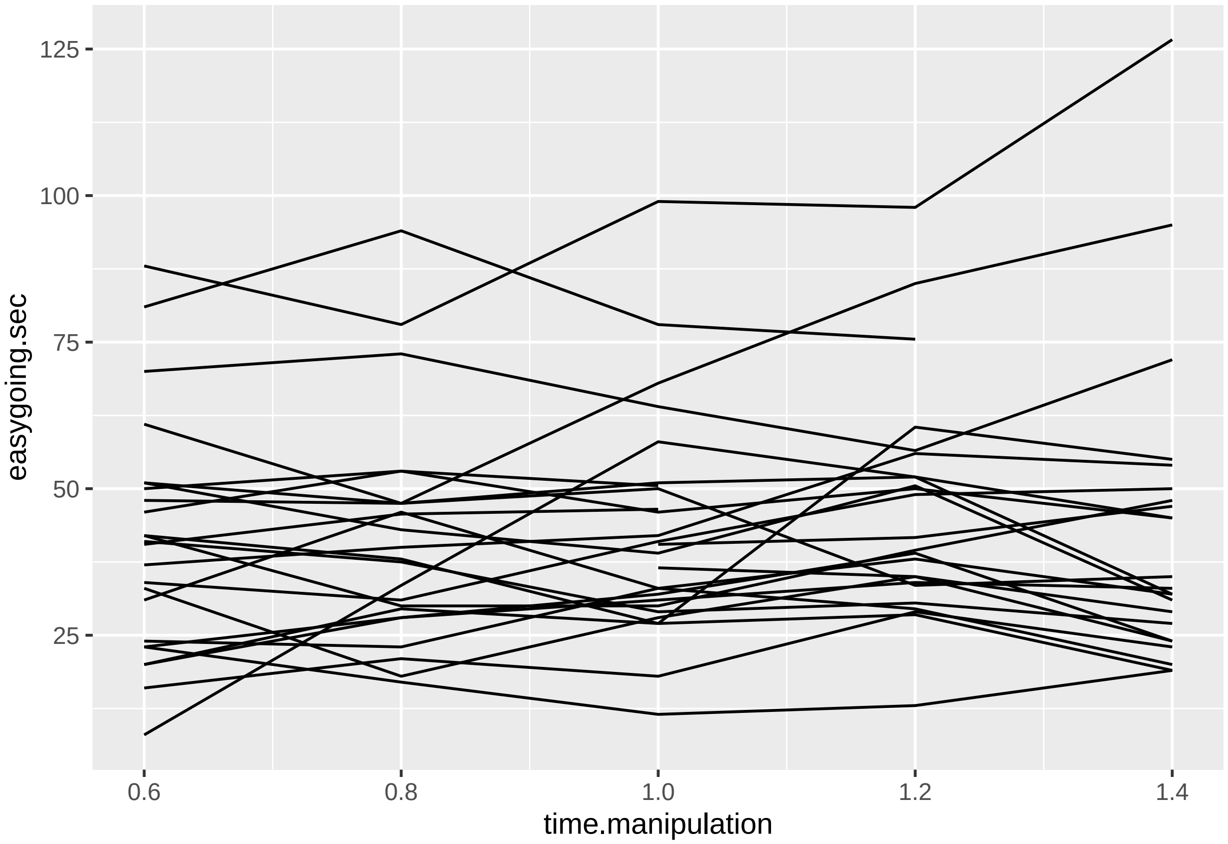

Set up the stats models. Same as overall apnea duration, but for the normalized easygoing duration.

```
lm.easy <- list()
lm.easy[["base"]] <- lme(easygoing.sec ~ trial.num,
              random = ~1 | individual,
              na.action = na.omit,
              method="ML",
              data = apneatrials.unaware)
lm.easy[["time"]] <- lme(easygoing.sec ~ trial.num + time.manipulation,
              random = ~1 | individual,
              na.action = na.omit,
              method="ML",
              data = apneatrials.unaware)
lm.easy[["full"]] <- lme(easygoing.sec ~ trial.num * time.manipulation,
              random = ~1 | individual,
              na.action = na.omit,
              method="ML",
              data = apneatrials.unaware)
```

```r
anova(lm.easy[["base"]], lm.easy[["time"]], lm.easy[["full"]])
```

```
##                    Model df      AIC      BIC    logLik   Test  L.Ratio
## lm.easy[["base"]]     1  4 1358.531 1371.098 -675.2657
## lm.easy[["time"]]     2  5 1356.303 1372.012 -673.1516 1 vs 2 4.228109
## lm.easy[["full"]]     3  6 1352.797 1371.647 -670.3986 2 vs 3 5.505955
##                    p-value
## lm.easy[["base"]]
## lm.easy[["time"]]  0.0398
## lm.easy[["full"]]  0.0190
```

Here, it looks like the interaction of trial and time manipulation matters. Let's look at this further.

```r
summary(lm.easy[["full"]])
```

```
## Linear mixed-effects model fit by maximum likelihood
##  Data: apneatrials.unaware
##        AIC      BIC    logLik
##   1352.797 1371.647 -670.3986
##
## Random effects:
##  Formula: ~1 | individual
##         (Intercept) Residual
## StdDev:    17.48375 9.628961
##
## Fixed effects: easygoing.sec ~ trial.num * time.manipulation
##                                Value Std.Error  DF   t-value p-value
## (Intercept)                18.918528  7.850144 142  2.409959  0.0172
## trial.num                   4.766336  1.711998 142  2.784078  0.0061
## time.manipulation          21.504477  7.144780 142  3.009817  0.0031
## trial.num:time.manipulation -3.920059  1.674269 142 -2.341355  0.0206
##  Correlation:
##                             (Intr) trl.nm tm.mnp
## trial.num                   -0.804
## time.manipulation           -0.872  0.862
## trial.num:time.manipulation  0.798 -0.973 -0.905
##
## Standardized Within-Group Residuals:
##         Min          Q1         Med          Q3         Max
## -2.61827757 -0.59974692  0.04856393  0.52974206  3.28187374
##
## Number of Observations: 171
## Number of Groups: 26
```

```r
emmeans(lm.easy[["full"]], ~time.manipulation,
        at = list(time.manipulation = seq(0.6, 1.4, by=0.2), trial.num = c(5)))
```

```
## NOTE: Results may be misleading due to involvement in interactions

##  time.manipulation emmean   SE df lower.CL upper.CL
##                0.6   43.9 3.92 25     35.8     52.0
##                0.8   44.3 3.69 25     36.7     51.9
##                1.0   44.7 3.58 25     37.3     52.0
##                1.2   45.0 3.63 25     37.6     52.5
##                1.4   45.4 3.81 25     37.6     53.3
```

```
##
## Degrees-of-freedom method: containment
## Confidence level used: 0.95
```

What this means is that the effect of time manipulation becomes less in later trials. Probably not an important point, particularly since not all individuals did the later trials.

```
plot(lm.easy[["time"]])
```

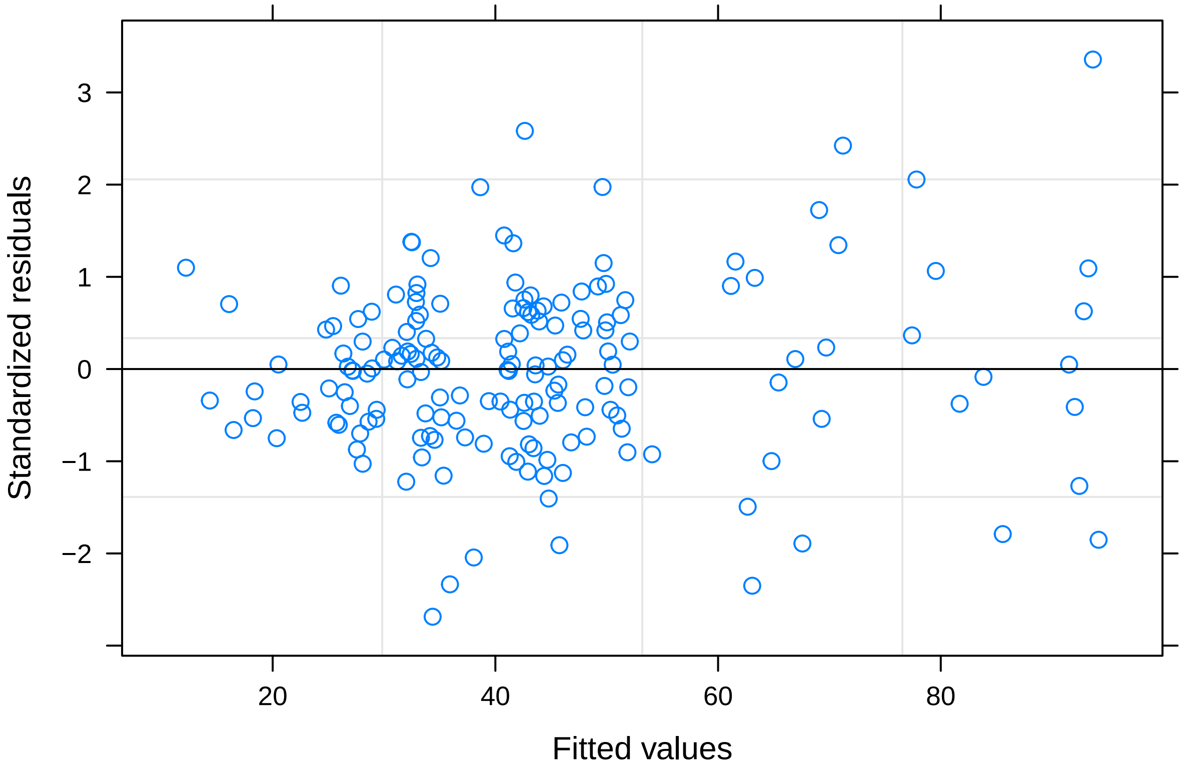

The variance seems to be a bit higher for longer durations, but it's not too extreme.

```
ggplot(data.frame(resid=residuals(lm.easy[["time"]])), aes(sample=resid)) +
  stat_qq_line() + stat_qq_band() + stat_qq_point() +
  labs(x = "Theoretical", y="Sample")
```

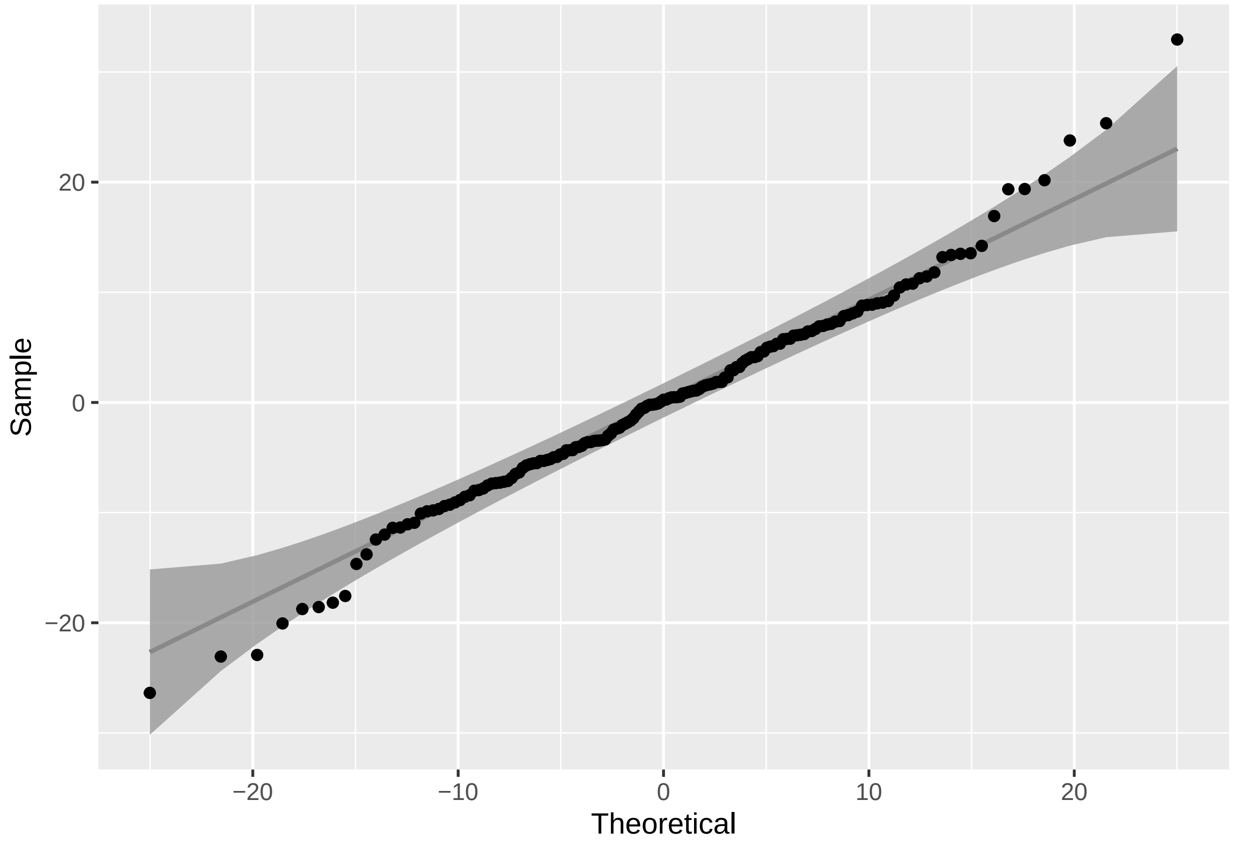

Looks quite good.

Get the predicted easygoing durations as a function of time manipulation.

```
lm.easy.fit <- as.data.frame(emmeans(lm.easy[["time"]], ~time.manipulation,
                            at = list(time.manipulation = seq(0.6, 1.4, by=0.2))))
```

Struggle phase

```
lm.struggle <- list()
lm.struggle[["base"]] <- lme(struggle.sec ~ trial.num,
              random = ~1 | individual,
              na.action = na.omit,
              method="ML",
              data = apneatrials.unaware)
lm.struggle[["time"]] <- lme(struggle.sec ~ trial.num + time.manipulation,
              random = ~1 | individual,
              na.action = na.omit,
              method="ML",
              data = apneatrials.unaware)
lm.struggle[["full"]] <- lme(struggle.sec ~ trial.num * time.manipulation,
              random = ~1 | individual,
              na.action = na.omit,
              method="ML",
              data = apneatrials.unaware)
```

```
anova(lm.struggle[["base"]], lm.struggle[["time"]], lm.struggle[["full"]])
```

```
##                         Model df       AIC       BIC     logLik    Test   L.Ratio
```

```
## lm.struggle[["base"]]     1  4 1343.917 1356.483 -667.9582
## lm.struggle[["time"]]     2  5 1344.143 1359.852 -667.0716 1 vs 2 1.773214
## lm.struggle[["full"]]     3  6 1344.575 1363.425 -666.2876 2 vs 3 1.568069
##                         p-value
## lm.struggle[["base"]]
## lm.struggle[["time"]]  0.1830
## lm.struggle[["full"]]  0.2105
```

Very little is significant for the duration of the struggle phase, but we'll stick with the same model.

```r
summary(lm.struggle[["time"]])
```

```
## Linear mixed-effects model fit by maximum likelihood
##  Data: apneatrials.unaware
##        AIC      BIC    logLik
##   1344.143 1359.852 -667.0716
##
## Random effects:
##  Formula: ~1 | individual
##         (Intercept) Residual
## StdDev:    21.13928 9.109862
##
## Fixed effects: struggle.sec ~ trial.num + time.manipulation
##                         Value Std.Error  DF  t-value p-value
## (Intercept)       12.222208  5.166578 143 2.365629  0.0193
## trial.num          0.540512  0.371514 143 1.454892  0.1479
## time.manipulation  3.809753  2.876696 143 1.324350  0.1875
##  Correlation:
##                   (Intr) trl.nm
## trial.num         -0.170
## time.manipulation -0.504 -0.186
##
## Standardized Within-Group Residuals:
##         Min          Q1         Med          Q3         Max
## -2.57996880 -0.55742046 -0.09298095  0.41280801  4.46048196
##
## Number of Observations: 171
## Number of Groups: 26
```

```r
plot(lm.struggle[["time"]])
```

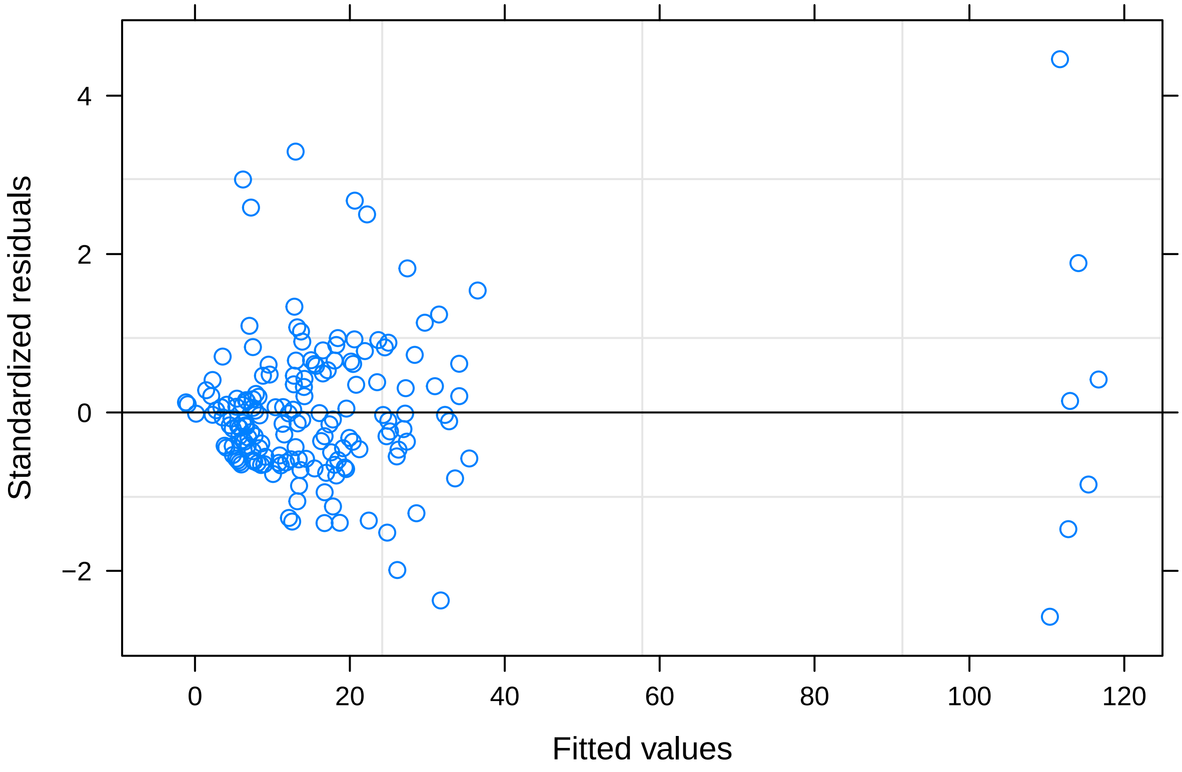

The results might be skewed by the one individual who struggled for a very long time.

```
lm.struggle[["minuslong"]] <- lme(struggle.sec ~ trial.num + time.manipulation,
              random = ~1 | individual,
              na.action = na.omit,
              method="ML",
              data = filter(apneatrials.unaware, struggle.sec < 100))
```

```
summary(lm.struggle[["minuslong"]])
```

```
## Linear mixed-effects model fit by maximum likelihood
##  Data: filter(apneatrials.unaware, struggle.sec < 100)
##        AIC      BIC    logLik
##   1265.293 1280.852 -627.6463
##
## Random effects:
##  Formula: ~1 | individual
##         (Intercept) Residual
## StdDev:    16.46308 8.223971
##
## Fixed effects: struggle.sec ~ trial.num + time.manipulation
##                      Value Std.Error  DF  t-value p-value
## (Intercept)       11.964984  4.287817 138 2.790461  0.0060
## trial.num          0.539897  0.339975 138 1.588049  0.1146
## time.manipulation  3.014509  2.632897 138 1.144940  0.2542
##  Correlation:
##                   (Intr) trl.nm
## trial.num         -0.201
## time.manipulation -0.559 -0.158
##
## Standardized Within-Group Residuals:
```

```
##          Min           Q1          Med           Q3          Max
## -2.61809006 -0.58719483 -0.09310571  0.43093293  3.64732149
##
## Number of Observations: 166
## Number of Groups: 26
```

But still nothing significant.

```
ggplot(data.frame(resid=residuals(lm.struggle[["time"]])), aes(sample=resid)) +
  stat_qq_line() + stat_qq_band() + stat_qq_point() +
  labs(x = "Theoretical", y="Sample")
```

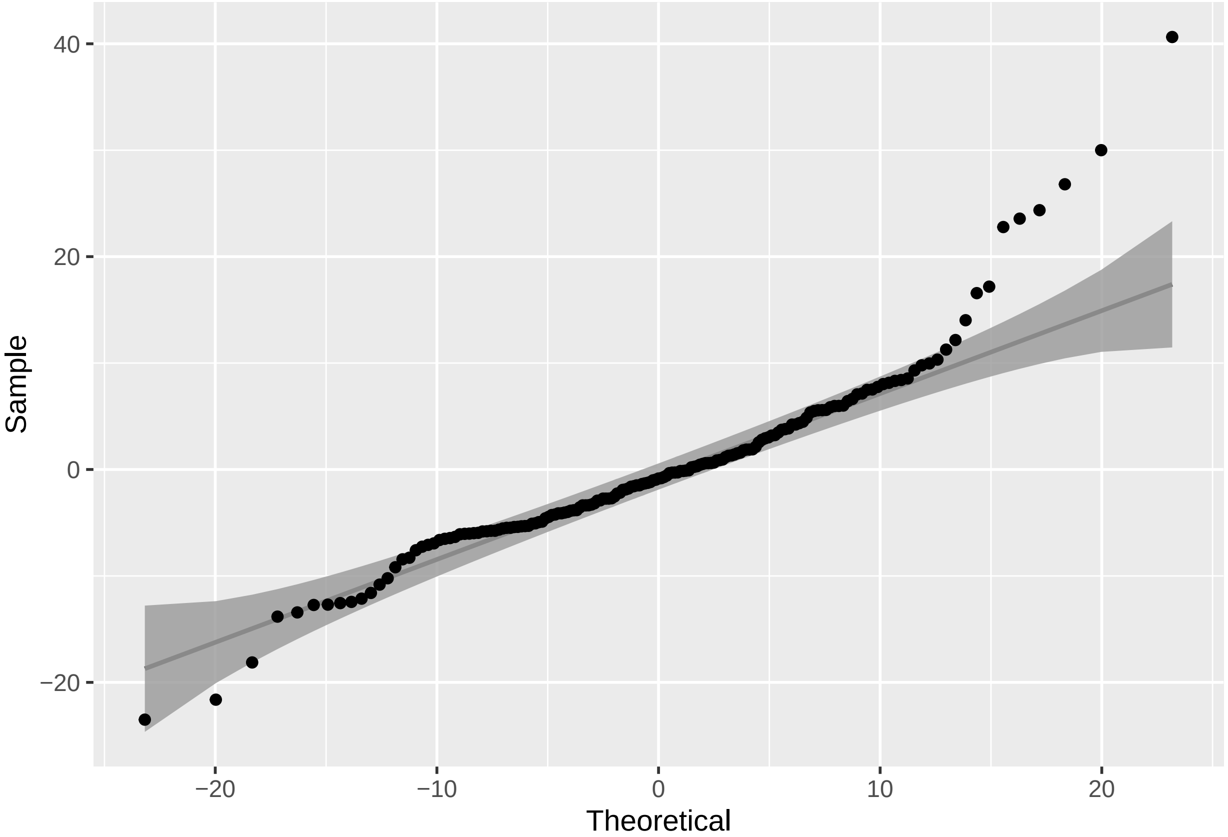

Long tail on the right.

```
lm.struggle.fit <- as.data.frame(emmeans(lm.struggle[["time"]], ~time.manipulation,
                            at = list(time.manipulation = seq(0.6, 1.4, by=0.2))))
```

Compare overall duration, easygoing and struggle phases

```
p1 <- apneatrials.unaware %>%
  group_by(individual, time.manipulation) %>%
  summarize(apnea.duration.sec = mean(apnea.duration.sec, na.rm=TRUE)) %>%
  ggplot(aes(x = time.manipulation, y = apnea.duration.sec)) +
  geom_line(aes(group=individual), size=0.5, color=gray) +
  geom_ribbon(data=lm.dur.fit, aes(x=time.manipulation, ymin=emmean-SE, ymax=emmean+SE), alpha=0.4,
              inherit.aes = FALSE) +
  geom_line(data=lm.dur.fit, aes(x=time.manipulation, y=emmean), inherit.aes = FALSE, size=1.5) +
  # geom_abline(slope=1, intercept=0, color="black", linetype=3, size=1) +
  labs(x="Time manipulation", y="Apnea duration (sec)") +
```

```
  ylim(0, 150) +
  papertheme + theme(panel.grid.minor.x = element_blank())

p2 <- apneatrials.unaware %>%
  group_by(individual, time.manipulation) %>%
  summarize(easygoing.sec = mean(easygoing.sec, na.rm=TRUE)) %>%
  ggplot(aes(x = time.manipulation, y = easygoing.sec)) +
  geom_line(aes(group=individual), size=0.5, color=gray) +
  geom_ribbon(data=lm.easy.fit, aes(x=time.manipulation, ymin=emmean-SE, ymax=emmean+SE),
            alpha=0.4, inherit.aes = FALSE) +
  geom_line(data=lm.easy.fit, aes(x=time.manipulation, y=emmean), inherit.aes = FALSE, size=1.5) +
  labs(x="Time manipulation", y="Easygoing phase duration (sec)") +
  ylim(0, 150) +
  papertheme + theme(panel.grid.minor.x = element_blank())

p3 <- apneatrials.unaware %>%
  group_by(individual, time.manipulation) %>%
  summarize(struggle.sec = mean(struggle.sec, na.rm=TRUE)) %>%
  ggplot(aes(x = time.manipulation, y = struggle.sec)) +
  geom_line(aes(group=individual), size=0.5, color=gray) +
  geom_line(data=lm.struggle.fit, aes(x=time.manipulation, y=emmean),
           inherit.aes = FALSE, size=1, linetype="dashed") +
  labs(x="Time manipulation", y="Struggle phase duration (sec)") +
  ylim(0, 150) +
  papertheme + theme(panel.grid.minor.x = element_blank())

fig.duration <- plot_grid(p1, p2, p3, nrow=1,
                          labels="AUTO", label_x = 0.18)
fig.duration
```

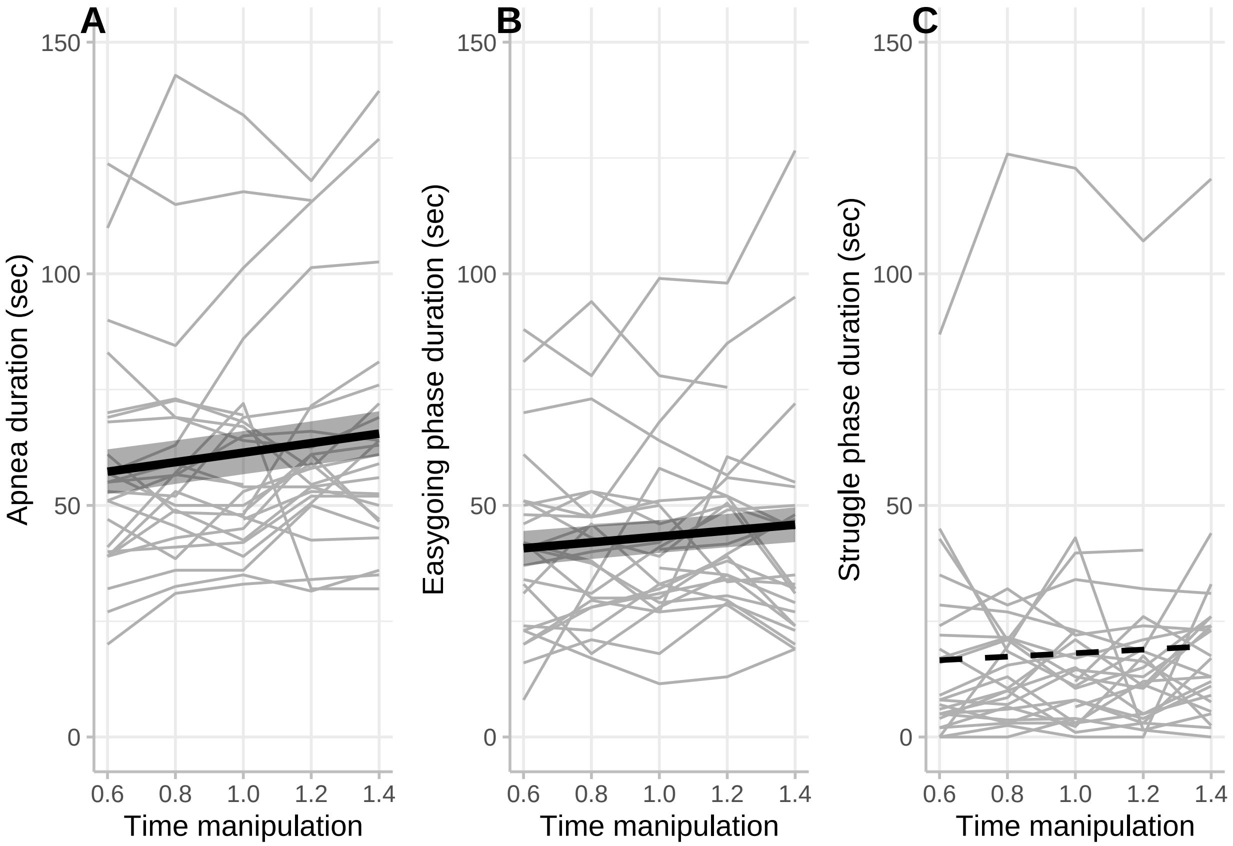


```r
filename <- 'Fig-duration.pdf'
ggsave(filename, plot=fig.duration,
       width=6.3,
       height=2.5, units="in", useDingbats=FALSE)
knitr::include_graphics(filename)
```

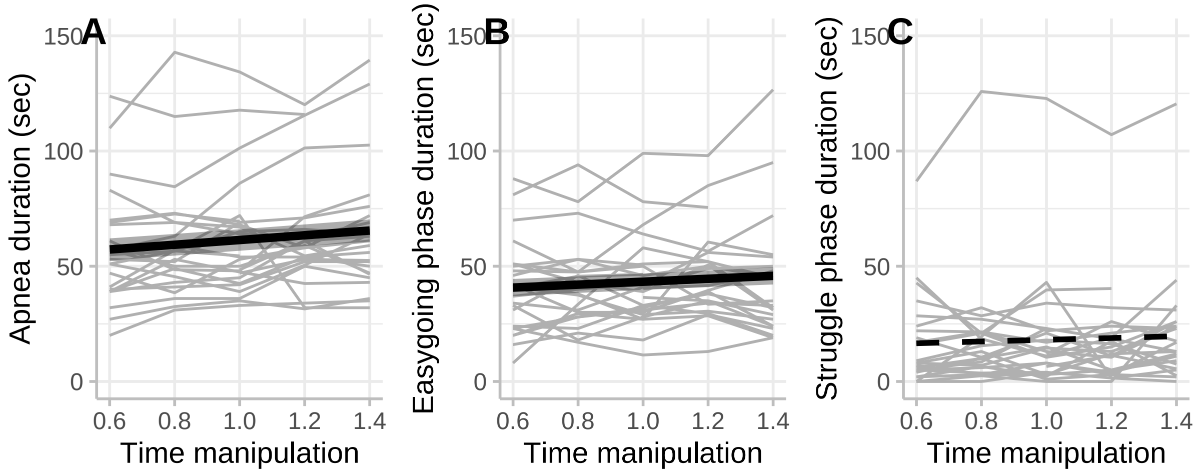

Fig. 5. Time manipulation affects the overall apnea duration and the easygoing phase duration, but not the struggle phase. Blue solid line, no apnea experience; green dashed lines, apnea experience; black dotted lines, individuals who became aware of the manipulation (only shown on panel A). Each line represents one individual. The fitted regression line is shown with a thick black line, and a 1:1 relationship is shown by a red line.

Generate stats tables

```
getanovacolumns <- function(lm) {
  df <- as.data.frame(anova(lm)) %>%
    rownames_to_column('effect') %>%
    transmute(effect=effect, F=`F-value`, df=sprintf("%d,%d", numDF, denDF), p=`p-value`) %>%
    column_to_rownames('effect')
  return(df)
}
```

```
getanovacolumns(lm.perception[["time"]])
```

```
##                              F    df           p
## (Intercept)       221.685417 1,143 0.000000000
## trial.num           2.469077 1,143 0.118316097
## time.manipulation  10.096479 1,143 0.001820627
```

```
statstable <- lapply(list(lm.perception[["time"]], lm.dur[["time"]],
                          lm.easy[["time"]], lm.struggle[["time"]]),
                     getanovacolumns)
statstable <- do.call(cbind, statstable)
```

```
kable(statstable, digits=rep(c(2,0,3), 5)) %>%
  kable_styling(full_width=FALSE) %>%
  add_header_above(c(" "=1, "Perception"=3, "Total duration"=3, "Easygoing phase"=3,
                     "Struggle phase"=3))
```

	Perception			Total duration			Easygoing phase			Struggle phase		
	F	df	p	F	df	p	F	df	p	F	df	p
(Intercept)	221.69	1,143	0.000	173.17	1,143	0.000	149.35	1,143	0.000	18.14	1,143	0.000
trial.num	2.47	1,143	0.118	17.54	1,143	0.000	6.72	1,143	0.011	3.00	1,143	0.085
time.manipulation	10.10	1,143	0.002	11.03	1,143	0.001	4.23	1,143	0.042	1.75	1,143	0.187

```
write.table(statstable, file="statstable.txt", sep='\t')
```
